# Integrative Infochemistry of Polyhydroxyalkanoate Films: Multimodal Data, Topological Tuning, and Cell–Surface Interfaces

**DOI:** 10.1021/acs.langmuir.6c02572

**Published:** 2026-08-22

**Authors:** Fares D. E. Ghorabe, Ashish T. S. Ireddy, Pavel S. Zun, Israel A. Huaman, Sviatlana A. Ulasevich, Aleksandr S. Aglikov, Dmitry A. Kozodaev, Tatiana G. Volova, Ekaterina I. Kessler Shishatskaya, Michael Nosonovsky, Ekaterina V. Skorb

**Affiliations:** † Infochemistry Scientific Centre, 65071 ITMO University, 9 Lomonosova Street, 191002 St. Petersburg, Russia; ‡ NT-MDT BV, 7335 Apeldoorn, The Netherlands; § 82505 Siberian Federal University, 79 Svobodnyi Av., Krasnoyarsk 660041, Russia; ∥ 104647 Institute of Biophysics SB RAS, Federal Research Center “Krasnoyarsk Science Center SB RAS”, Akademgorodok, 50/50, 660036 Krasnoyarsk, Russia; ⊥ 14751 University of Wisconsin-Milwaukee, Milwaukee, Wisconsin 53217, United States

## Abstract

This study investigates seeding and cultivation of C2C12 myoblast cells on poly­(3-hydroxybutyrate-*co*-3-hydroxyvalerate) (P3HBV) films synthesized at four polymer concentrations, yielding films with progressively increasing lateral thickness and diverse surface topographies. Cell attachment was evaluated after 24 h, alongside correlations between surface topography and cellular behavior. Surface characteristics across films at each thickness were quantified using atomic force microscopy (AFM), scanning electron microscopy (SEM), advanced topological data analysis (TDA), and threshold relief analysis. C2C12 myoblast growth was imaged via fluorescence microscopy, with quantitative metrics extracted using the Cellpose Plus toolbox. Statistical modeling revealed correlations between surface features and cell morphology. SEM imaging further corroborated pore characteristics against cell growth metrics. Results demonstrate a linear relationship between surface topography and cellular responses, underscoring the critical role of substrate morphology in modulating cell behavior, where films synthesized at high polymer concentrations (5–7% w/v) exhibit enhanced cell attachment. The 85:15 P3HBV copolymer was selected for its favorable thermomechanical properties, high biocompatibility, and cost-effectiveness for large-scale biosynthesis. Our work presents a reproducible data-driven pipeline for analyzing cell–surface interactions, combining TDA and image segmentation metrics to explore the behavior of surface morphology under varying material conditions.

## Graphical abstract

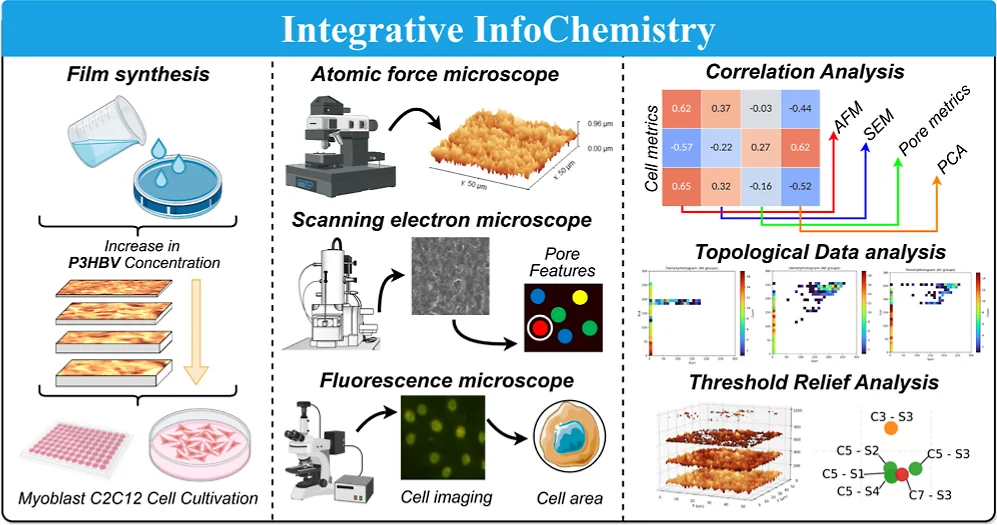

## 1 Introduction

Polymers have revolutionized materials science across modern industries through their versatility, biocompatibility, and tunable physicochemical properties. From established roles in drug delivery and medical devices to emerging applications in tissue engineering and regenerative medicine, both natural and synthetic polymers provide platforms that mimic the tissue extracellular matrix (ECM), exhibit controlled degradation, and support cell adhesion and proliferation.^1^ Among biodegradable polymers, microbially produced polyhydroxyalkanoates (PHAs) reserve carbon/energy storage compounds have gained prominence due to their total renewability, complete biodegradability into nontoxic end products, and excellent bio- and cytocompatibility.^2^ Produced by various procaryotes under nutrient-limiting conditions as cytoplasmic inclusions, PHAs degrade naturally into nontoxic bioproducts, making them ideal candidates for biomedical use. Within this family, poly­(3hydroxybutyrate-*co*-3hydroxyvalerate) (P3HBV) stands out for its improved thermomechanical properties, sufficient elasticity and biocompatibility compared to poly­(3-hydroxybutyrate) (P3HB).^3–6^

The biomedical potential of PHAs extends beyond bulk degradability to their tunable surface characteristics, which critically mediate cellular responses at the material–cell interface. Increasing evidence suggests that cells are extremely sensitive to both the chemical composition and the physical features of the substrate surface at the micro- and nanoscale.^7^ Parameters such as surface roughness (S_a_), morphology, texture, porosity, energetic characteristics and hydrophilicity/hydrophobicity, and the presence of some functional groups critically impact the initial protein adsorption profile and downstream cellular processes including adhesion, proliferation, migration, differentiation, and phenotype.^8,9^ Furthermore, the copolymer composition such as the ratio of 3-hydroxyvalerate (3HV), 4-hydroxybutyrate, or 3-hydroxyhexanoate (3HHx) units can significantly affect atomic and molecular packing during crystallization reflecting in surface chemistry, energy, and topography, thereby modulating specific cellular behaviors.^10^ An equally important yet less-explored factor is substrate thickness, which can modulate stiffness, durability, degradation rate, and mass transport properties. For instance, thinner films may allow some cells to detect and respond to the rigidity, influencing cell morphology, migration, and even lineage commitment.^11,12^ In contrast, thicker films create a more PHA dominant mechanical microenvironment, potentially altering cell behavior over time. Cell phenotype and mechanochemical behavior are crucial for applications of this material in implants and other biomedical applications.^2,13^ As these films degrade, thickness-dependent changes in surface chemistry and topography may further influence dynamic cellular responses and body reactivity in total.^14,15^ Despite these advances, a number of technical and analytical challenges remain in the systematic study of cell–polymer interactions. Considering the outstanding potential of PHAs as biopolymers in tissue reconstruction, drug design, and cell technologies, there is an urgent demand to elucidate structure–surface property relationships of PHAs. A major limitation is the interdependence of surface parameters such as chemistry, roughness, and thickness. Modifying one often affects others, making it difficult to isolate individual effects.^16^ The same PHA surface may also elicit varied responses from different cell types, complicating the establishment of universal guidelines. Moreover, film preparation methods (e.g., solvent casting, spin coating, deep coating, compression molding, or melt extrusion) can introduce batch-to-batch variability in surface morphology and crystallinity, limiting experimental reproducibility and cross-study comparisons.^17^ Analytical tools such as AFM, X-ray photoelectron spectroscopy (XPS), and contact angle goniometry are essential for quantifying nanoscale surface features, yet even minor inconsistencies in sample preparation can yield divergent results. Compounding this issue is the dynamic nature of cell–material interactions, where protein adsorption and matrix deposition by cells evolve over time, masking the original surface characteristics.^18^

An important step in our analysis is the identification of all cells and nuclei in the images. Traditionally, the cell image segmentation process relied on classical algorithms (e.g., thresholding and watershed) that are often sensitive to specific imaging conditions, noise, and cell density (confluence), requiring laborious manual optimization for each new experiment. The introduction of deep learning, particularly convolutional neural networks (CNNs), has dramatically improved the accuracy and generalizability of image segmentation. Among these tools, Cellpose,^19^ trained on a vast diversity of cell types and imaging conditions from various microscopes, offers accurate cell segmentation through a user-friendly interface, making it ideal for our purposes. Despite this powerful foundation, segmentation is just the starting point for the cell activity analysis. Researchers must still measure cell morphological characteristics (e.g., size and roundness), assess sample uniformity (e.g., via Voronoi tessellation), and perform statistical analyses across populations. This often requires a disjointed manual workflow across multiple software packages, introducing complexity and potential errors. To address this integration challenge, we employ Cellpose Plus,^20^ a comprehensive analytical framework built upon the Cellpose engine that unifies state-of-the-art segmentation with automated tools for quantifying key cellular behaviors.

In recent years, topological data analysis (TDA) has emerged as a powerful approach for processing AFM surface data, enabling the extraction of scale-invariant structural descriptors via persistence barcodes and diagrams with Notable applications include several works.^21–23^ These studies demonstrate TDA’s capability to reveal multiscale topographic features not captured by conventional roughness metrics. Also, in a prior experiment,^21^ we introduced a novel heuristic phenomenological model for analyzing the evolution of contact areas on rough surfaces across multiple deformation stages threshold relief analysis (TRA). By employing a cutoff-threshold filtration to track both numerical and topological metrics, such as distributions of contact-area subregions and self-affine parameters, this method achieves robust cross-scale characterization, as shown in a previous work.^24^ TDA and TRA were previously used to produce features for ANN (Artificial Neural Network) building. Yet there remains a lack of predictive approaches linking surface characteristics to cellular outcomes. Most studies are empirical, focusing on single cell type or narrow material parameters, which slows progress and limits the broader application of PHA-based biomaterials in clinical or industrial settings.^25^ Given these challenges and the rising interest in leveraging PHA copolymers for biotechnological innovation, a deeper understanding of how surface characteristics, particularly roughness, chemistry, and thickness, influence cellular responses is crucial. The goal of this work is to characterize PHA biomaterial surface features using SEM and AFM and then apply TDA and TRA to extract multiscale topological insights from these data sets. By correlating surface variations with early cell attachment behavior, we elucidate how topography governs structure–cell interactions for optimized biomedical PHA designs.

## 2 Materials and Methods

In this study, we investigated the influence of increasing P3HBV solution concentration on the physicochemical properties of synthesized polymer films, including thickness and surface characteristics, and their subsequent effects on early cellular attachment and mobility.^26^ P3HBV films were fabricated from solutions at four concentrations: 1%, 3%, 5%, and 7% (w/v). Different concentrations of P3HBV were dissolved in 10 mL of chloroform by using the solvent-casting method. Each polymer solution was poured into a clean 10 cm diameter Petri dish, cast inside a fume hood, and allowed to dry at room temperature for 48 h to ensure complete solvent evaporation (Figure S1 in the Supporting Information presents a complete overview of all acquired films). These concentrations were selected to explore how subtle changes in solution density affect surface topography, lateral thickness, and adhesive properties for cells in cast films. The surface morphology of the films was characterized using AFM. Acquired quantitative data were processed using the Gwyddion software toolbox[Fn fn1] ^27^, enabling the extraction of topographical features such as surface roughness (S_a_) parameters: average height, its standard deviation, skewness, and kurtosis (Table S1 in the Supporting Information provides a complete overview of extracted AFM topological features). To further analyze the spatial complexity and structure of the film surfaces, we employed TDA techniques on the surface data to calculate persistence homology and barcodes,^22^ which provide robust metrics of geometrical and topological variation beyond conventional surface roughness measurements and TRA (i.e., lacunarity measure)^24^ as high-dimensional descriptors for in-depth layered analysis of the surfaces.

To assess the biological relevance of these films, myoblast C2C12 cells were cultured directly on the polymer surfaces. To evaluate cells after establishment of stable attachment and spreading, cell behavior was assessed after 24 h of culture. Earlier time points (e.g., 6 h) were not selected because they mainly represent the initial adhesion stage, during which cell–material interactions are dominated by nonspecific physicochemical forces followed by protein-mediated integrin interactions and cytoskeletal reorganization. During this period, many cells remain partially spread or exhibit rounded morphology before formation of mature adhesion structures.^28^ A 24 h incubation period is commonly used in biomaterial studies to evaluate cell morphology, attachment, and spreading after sufficient time for focal adhesion development.^29^ Measurements were not performed after 48 h because extensive cell proliferation and surface coverage resulted in high confluence, preventing reliable evaluation of individual cell morphology and attachment characteristics. After incubation for 24 h, fluorescence microscopy was used to visualize cellular adhesion, morphology, and distribution. High-resolution images were segmented and analyzed using Cellpose Plus[Fn fn2] ^20^, an advanced deep learning-based image segmentation toolkit. From these images, a range of descriptive cell metrics were extracted, including cell roundness, nucleus size and area, total cell area, and cell confluence and density, key indicators of cellular viability, adhesion, and proliferation. Further, SEM was used to acquire the surface images of P3HBV films to assess surface porosity. Together, these cellular descriptors were then integrated with AFM-derived surface data and topological features to establish correlations between surface properties and cell activity. Statistical analysis was performed to identify trends and build a data-driven framework for understanding how P3HBV film characteristics modulate biological responses.

### 2.1 Instrumental and Mathematical Methods

The surface roughness of the films was evaluated using AFM in semicontact mode. Measurements were conducted under standard conditions using an NTEGRA ACADEMY AFM system (NT-MDT, Russia). An NSG01 cantilever, featuring a tip curvature radius of 8–10 nm, was employed for the scans. To ensure representative and accurate surface profiling, several regions measuring 50 × 50 μm were selected for analysis. High-resolution scanning at 256 × 256 pixels was used to capture fine surface details. Postprocessing of AFM images was performed using the Gwyddion toolbox,^27^ for artifact correction and enhancement of data quality via operations such as plane leveling, facet leveling, alignment of rows, and horizontal scar removal. To ensure the optimal performance of the AFM system, all measurements were conducted in a clean, vibration-isolated environment with an ISO cleanliness class of at least 6 or 7. Ambient conditions were strictly controlled, with a temperature range maintained between 20 and 25 °C, relative humidity at 20–30%, and atmospheric pressure around 760 mmHg, to prevent drift and moisture-related interference during scanning. From this data, we extracted the average surface value, surface roughness (S_a_), skewness (S_sk_) (measure of the asymmetry), and excess kurtosis (S_ku_) (measure of surface peaks). The data were evaluated for samples individually, and the average for all films within a concentration was also extracted. To ensure consistency, we refer to films of increasing thickness synthesized with concentrations of 1, 3, 5, and 7% as C1, C3, C5, and C7, as shown in Table 1.

**Table 1:** Characteristic Properties of P3HBV Films [C1, C3, C5, C7], AFM, SEM, and Fluorescence Microscopy, along with Cellpose Plus Metrics Averaged across Individual Samples per Group

metric	source	C1	C3	C5	C7
solution composition	experimental	0.1 g in 10 mL, 1% P3HBV	0.3 g in 10 mL, 3% P3HBV	0.5 g in 10 mL, 5% P3HBV	0.7 g in 10 mL, 7% P3HBV
film thickness (in μm)		13 ± 3	30 ± 9	49 ± 17	76 ± 14
surface roughness (S_a_)	AFM	88 ± 2	299 ± 24	379 ± 24	560 ± 87
skewness(S_ku_)		0.05 ± 0.05	–0.30 ± 0.12	–0.40 ± 0.20	–0.45 ± 0.26
excess kurtosis(S_ku_)		0.06 ± 0.1	0.40 ± 0.1	0.66 ± 0.32	0.70 ± 0.18
mean number of Pores	SEM	650 ± 11	784 ± 157	265 ± 18	388 ± 64
average total pore area (μm^2^)		391 ± 23	1324 ± 50	1070 ± 299	1129 ± 93
mean pore area per μm^2^		0.05 ± 0.01	0.16 ± 0.01	0.13 ± 0.03	0.14 ± 0.01
cell count	fluorescence microscope (Cellpose Plus)	62 ± 24	56 ± 20	142 ± 54	149 ± 54
cells per mm^2^		214 ± 82	194 ± 70	489 ± 187	513 ± 184
confluence, %		14 ± 6	8 ± 3	24 ± 9	34 ± 19
cell to nuclei area ratio		3.7 ± 0.6	3.2 ± 0.2	3.9 ± 0.5	4.2 ± 0.5
average cell size (μm^2^)		642 ± 71	391 ± 29	503 ± 31	623 ± 134
average nucleus size (μm^2^)		174 ± 12	128 ± 9	142 ± 13	148 ± 14
cell roundness		0.61 ± 0.04	0.68 ± 0.04	0.69 ± 0.04	0.72 ± 0.03
nuclei diameter (μm)		7.4 ± 0.3	6.3 ± 0.2	6.7 ± 0.3	6.9 ± 0.3
Voronoi entropy		1.54 ± 0.1	1.63 ± 0.1	1.57 ± 0.1	1.57 ± 0.1

To obtain greater insight into surface characterization, SEM was performed using an FEI Quanta Inspect microscope. Before SEM imaging, films were coated with gold to provide good conductivity. Operating with a beam voltage in the range of 10–20 kV, high vacuum mode 6 × 10^–4^ Pa, and a beam current exceeding 2 μA, the microscope provides high-resolution imaging and elemental analysis. A complete overview of acquired SEM images is shown in Figures S7 and S8 in the Supporting Information.

### 2.2 Cell Staining

After incubation in the presence of films, the cells were washed twice with PBS (pH 7.4) to remove nonadherent cells. Fixation was carried out using 95% cold ethanol by incubating the cells at 4 °C for 10 min, followed by three PBS washes. Permeabilization was not required due to the ethanol-based fixation. For viability-based cytoplasmic and nuclear staining, respectively, the cells were incubated with a dual-staining solution containing Acridine Orange (AO, 0.5 μg/mL) and Propidium Iodide (PI, 1 μg/mL) in PBS for 10 min at room temperature in the dark. AO produces green fluorescence of the cytoplasm and nuclei, while PI penetrates cells with compromised membranes, resulting in red fluorescence of the nuclei. After staining, the cells were washed gently with PBS to remove excess dye and immediately observed under a fluorescence microscope using appropriate filters for AO (green) and PI (red) fluorescence. Figure S9 in the Supporting Information provides an overview of acquired cell images from a fluorescence microscope. We used a model culture with outstanding viability to get cell growth images fast and reliably.

### 2.3 Cell Adhesion Analysis Using Cellpose Plus

For this step, we perform image segmentation, the task of identifying each individual cell and/or nucleus within an image. This is done using the Cellpose Plus^20^ software package. On acquiring individual fluorescence images from cells and nuclei, our first step is to join both images, taking individual color channels (green and blue, respectively). For the segmentation of cells and nuclei, we used pretrained models *cyto3* and nuclei based on U-net and implemented algorithms for convex hull,^30^ Voronoi diagram,^31^ Voronoi entropy,^32^ and Continuous Symmetry Measure (CSM)^33^ generation to evaluate the cells’ spreading behavior and density over the surface. We also analyzed individual cell and nucleus properties such as roundness (computed as $\frac{4\pi A}{P^{2}}$ , where *A* is the area and *P* is the perimeter) and area (μm^2^), as well as the area ratio between each cell and its nucleus. The results generated were exported to (.csv) files and were then used in our analysis. Table S5 in Supporting Information contains the extracted metric results for all captured cell images.

### 2.42.4 Topological Data Analysis

TDA is a powerful method of data analysis which reveals topological structure of data sets (e.g., surfaces to which data points tend to adhere in multidimensional dataspaces) and how this structure depends on scale.^2,21–24^ For the TDA analysis, we applied the Vietoris–Rips filtration approach over latent space builder on relief height map with the construction of persistence barcodes and diagrams (shown in the Supporting Information Figure S2 and Tables S2 and S3), as described in our prior methodological work.^34^ This method enables quantitative characterization of nanoscale AFM surface topographies through computation of persistence homology, bottleneck and 1-Wasserstein (earth mover’s) distances between persistence diagrams, and related statistical descriptors. Using a custom script, we calculated the 1-Wasserstein and bottleneck distances. The 1-Wasserstein distance (i.e., earth mover’s metric) provides a measure of dissimilarity between two probability distributions and quantitatively represents the level of dissimilarity between persistence diagrams of each P3HBV instance. The bottleneck distance is a metric to quantify the most significant discrepancy between two distributions. It measures and quantifies the dissimilarity between persistence diagrams and provides the minimum cost required to match significant features between them. Together, these metrics provide a compact and structured overview of the surface behavior of P3HBV films [C1, C3, C5, C7] and were henceforth used to establish and confirm the impact on surface topology.

Next, we employed a height-threshold filtration TRA method with subsequent calculation of numerical and topological metrics (e.g., number and area of first- and second-order objects, fractal dimension, and lacunarity). The method is calibration-free and scale-invariant, as demonstrated on AFM, SEM, synthetic, and Earth geodetic surface data sets.^24^ In this approach, the algorithm acquires slices of the surface in the *Z* axis (i.e., height) of the AFM film in equidistance thresholder steps, where each slice is binarized using the Bradely Roth approach and its surface features are analyzed to reveal lands (contact region) and holes (noncontact regions). Next, these lands and holes are analyzed to reveal the surface feature as a fractal lacunarity measure. Within this approach, the lands and holes can be counted in two ways, as first- and second-order box counting approaches, where the first-order lands are considered islands and second-order lands are considered nested islands, and the same is applied for holes, with inversion of concept (i.e., holes and nested holes). Further, the level of detail (i.e., neighborhood contact area) during threshold filtration can be implemented in either a 4-neighborhood or 8-neighborhood counting approach. Within our work, we follow an exhaustive approach for both neighborhoods and land–hole counting approaches to ensure a consistent and complete analysis of the data. In total, we had 12 combinations of feature control that consisted of three threshold steps of [100, 500, 1000] in [4, 8] neighborhood areas assessed over first- and second-order box counting [i.e., 3 × 2 × 2]. Upon completion of one iteration of TRA, we acquire a (.csv) file with a numerical count of the number of features (holes, internal lands, external lands, and internal holes), recorded over each slice (Figures S3–S6 in the Supporting Information provides a complete overview of feature-based statistics across C1 – C7 films).

## 3 Results and Discussions

The pipeline of the study is highlighted in Figure 1. The P3HBV copolymer was synthesized through pilot-scale fed-batch fermentation, followed by a multistep extraction and purification process to obtain a pure polymer powder. Ion exchange chromatography–mass spectroscopy confirmed the copolymer composition as P3HBV with an 85:15 molar ratio of 3-hydroxybutyrate to 3-hydroxyvalerate monomers.

**Figure 1:**
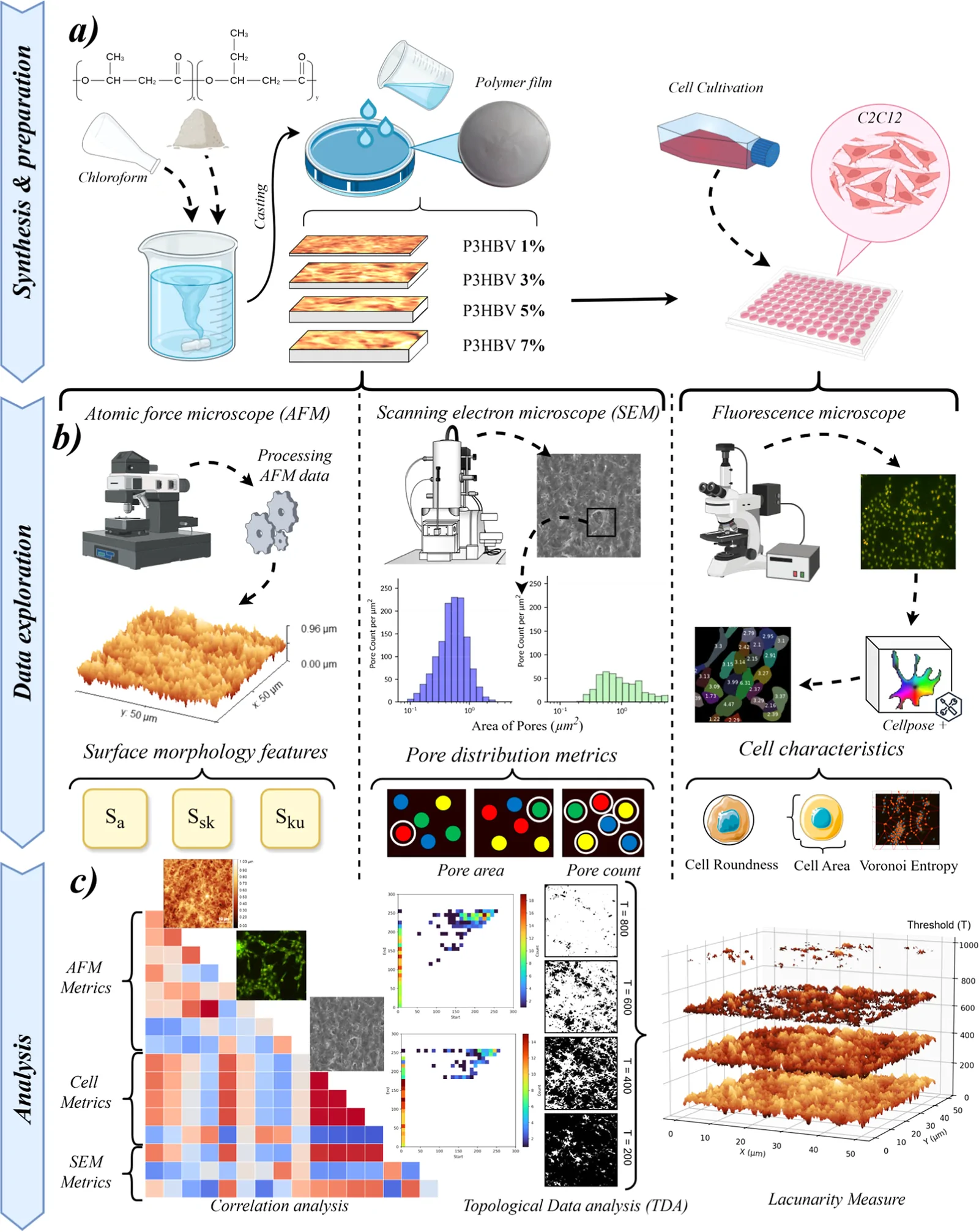
Overview of PHA surface topography effects on myoblast cell attachment and analytic pipeline: (a) P3HBV solutions prepared at concentrations of 1, 3, 5, and 7%, were deposited with the solvent casting method to obtain films of increasing thicknesses. (b) Casted P3HBV films were scanned using AFM and SEM, processed with Gwyddion and analyzed with Cellpose Plus, and then their surface features were extracted; cells were cultured on the upper side of cast films, and their images were captured with a fluorescence microscopy, marking the cytoplasm and nuclei. Segmentation and extraction of cell features were performed with the Cellpose Plus toolbox. (c) Analysis of data and metrics from an atomic force microscope, scanning electron microscopy, and fluorescence microscopy was performed using TDA, lacunarity measure to study correlative behavior, and dependency between surface features and C2C12 cell adhesion and growth.

Gel permeation chromatography analysis revealed a weight-average molecular weight (*M*_w_) of 690 kDa and a polydispersity index (D̵) of 2.8, indicating a normal molecular weight distribution for batch culture. Thermal characterization revealed a melting temperature (*T*_m_) of 165 °C and an onset of thermal degradation at approximately 275 °C.^35^ The copolymer was processed into thin samples via solvent casting, resulting in flexible, transparent films of increasing thickness (as shown in Table 1 and Figure 2A) with well-developed surface morphology and minimal porosity, as observed by SEM in Figure 2B. We synthesized a minimum of three films under each thickness corresponding to the concentration of P3HBV, denoted in the article as C1– S1, C1– S2, etc. (i.e., Concentration 1–Sample 1) and similarly for C3, C5, and C7 to ensure consistency of surface properties. Figure S1 in the Supporting Information provides a complete overview of all our films. Next, we cultivated C2C12 cells on the upper side of the respective films. Once completed, we used fluorescence microscopy to scan the cell growth over these films after 24 h (Figure 2C) to ensure consistent focal adhesion of the cells on the film surface. For each P3HBV film (C1, C3, C5, C7), we acquired 5 cell images capturing different areas of interest in the synthesized C2C12 regions. Next, we used the Cellpose Plus toolbox to segment and generate cell adhesion metrics for analysis. We extracted features describing cell condition using Cellpose Plus metrics, as shown in Table 1.

**Figure 2:**
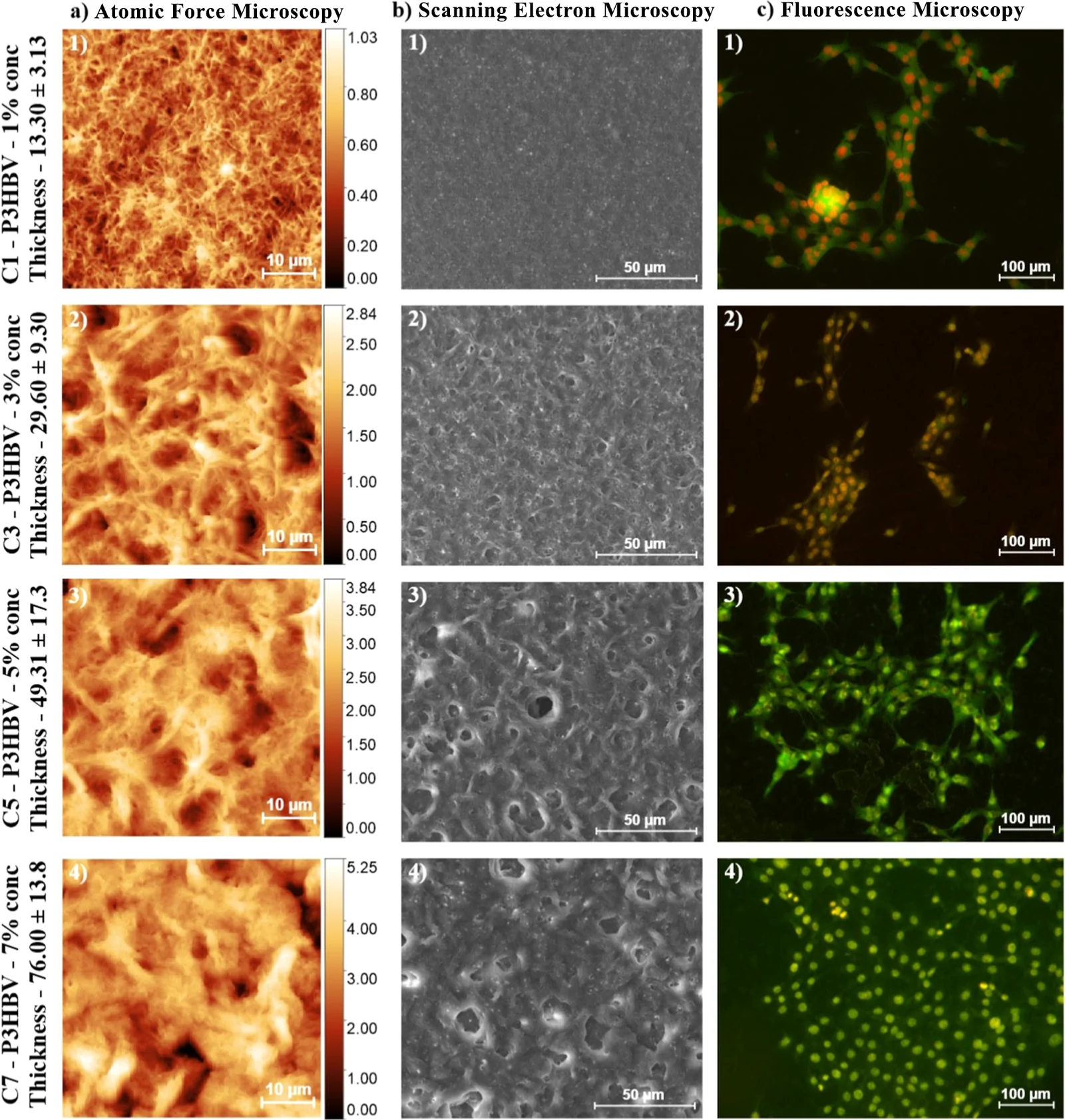
Column (a) (1–4): AFM images of the P3HBV films produced from 1%, 3%, 5%, and 7% P3HBV solutions; column (b) (1–4): SEM images of the P3HBV films corresponding to concentrations 1% to 7%; column (c) (1–4): fluorescence microscopy images of the myoblast C2C12 cells cultivated on respective films for 24 h shown in increasing concentration from C1:1% P3HBV to C7:7% P3HBV.

Analysis of AFM data and its surface characteristics shows a concentration-dependent trend where an increase in polymer concentration yields thicker films with a progressive rise in surface roughness. The surface skewness (S_sk_) and excess kurtosis (S_ku_) are also observed to shift with a change in film thickness. Thin films (with a 1% solution concentration of P3HBV) (C1) are dominated by protrusions and peaks, whereas thicker films (with a 7% solution concentration of P3HBV) (C7), exhibiting predominantly negative skewness, indicate a surface profile enriched in depressions, trenches, and valleys, as shown in Figure 3. In parallel, kurtosis increases with film thickness, consistent with the emergence of sharper, more pronounced surface features; this behavior follows through with the skewness shift toward deeper trough-like morphologies. The transition from positive to negative skewness occurred monotonically with increasing polymer concentration and corresponding film thickness, with average Ssk values changing from 0.05 ± 0.05 (C1) to −0.30 ± 0.12 (C3), −0.40 ± 0.20 (C5), and −0.45 ± 0.26 (C7). No nonmonotonic behavior was observed for the intermediate concentrations. Upon culturing C2C12 cells on these films, we observe two dominant cluster-based characteristics such that the thicker (higher-concentration) films support higher cell growth and metabolic activity, implying that the more developed topography and associated thickness-related changes provide a more favorable interface for cell spreading and proliferation, an effect broadly consistent with the known sensitivity of cells to micro/nanoscale surface features. This relationship between processing (casting concentration), resulting film structure (thickness/morphology), and cellular response is practically important for designing PHA-based surfaces where robust cell adhesion and growth are desired for example, in tissue-engineering scaffolds or surface-engineered biomedical devices (adapt as needed for bone-regeneration scaffolds, vascular grafts, or implant coatings).

**Figure 3:**
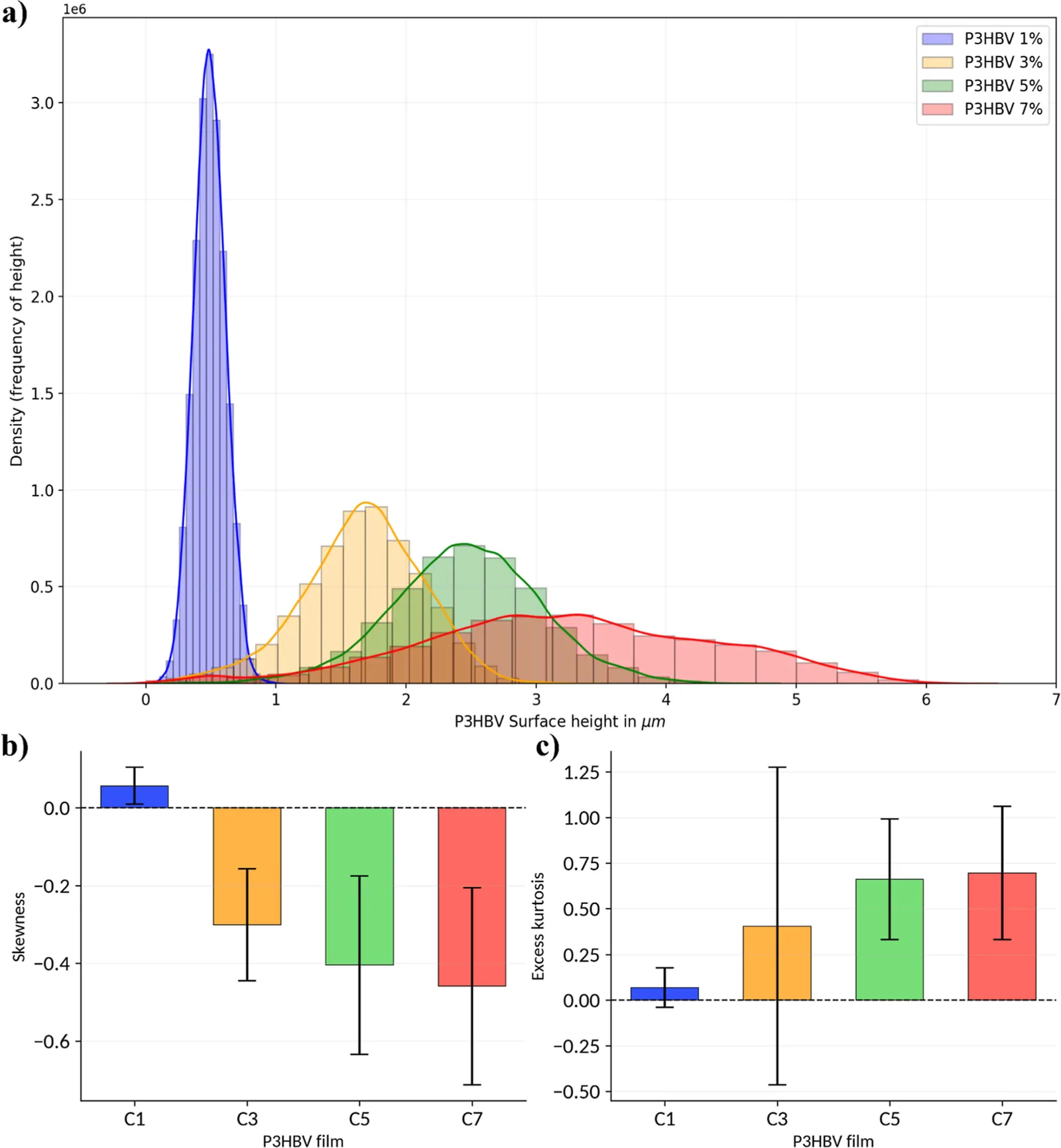
Observed surface characteristics of produced P3HBV films [C1, C3, C5, C7]. (A) Distribution of surface heights over AFM films; (B) surface skewness (S_sk_); and (C) excess kurtosis distribution (S_ku_).

Fluorescence microscopy analysis on data from the Cellpose Plus toolbox and AFM revealed distinct correlations between film properties and cellular adhesion. First, cell growth (total cell count) increased with polymer concentration and film thickness, as shown in Table 1. This trend correlates strongly with surface topography; specifically, cell attachment and proliferation were enhanced on surfaces exhibiting higher roughness, negative skewness (S_ku_) (predominance of trenches/valleys), and lower kurtosis (S_ku_) (indicative of wider features). This suggests that thicker films, which possess coarser and more cavernous topographies, provide a superior microenvironment for cell colonization compared with the smoother, peak-dominated surfaces of thinner films. This is because thicker films cast from higher concentration solutions undergo slower solvent evaporation, allowing P3HBV to crystallize into larger, stiffer lamellar structures that create a rougher, trench-enriched topography.

The cell layer confluence (i.e., the total area covered by cells in the film) increased in parallel with film thickness. Notably, the quality of cell confluence appears driven by specific topographical features: we note large areas of P3HBV films, totally covered with cell layers; obviously, this phenomenon was associated with surfaces characterized by negative skewness (more trenches) and lower kurtosis (wider features). However, unlike cell count, the cell-covered area showed no direct correlation with global roughness or film thickness alone, suggesting that it is regulated more by local feature geometry (trenches) than average roughness. Nucleus area (i.e., area covered by nuclei) mirrored cell-covered area trends but displayed a unique positive correlation with both surface roughness and film thickness, indicating that nuclear morphology may be more sensitive to global surface parameters than the cell area, Figure 4. We noted some decrease in cell spreading with increasing thickness, and cells became slightly more spherical. As these results demonstrate, we can empirically approach the optimal substrate for each cell culture; however, we cannot yet numerically express the mechanical connection between the polymer stiffness and the local membrane deformation at contact sites.

**Figure 4:**
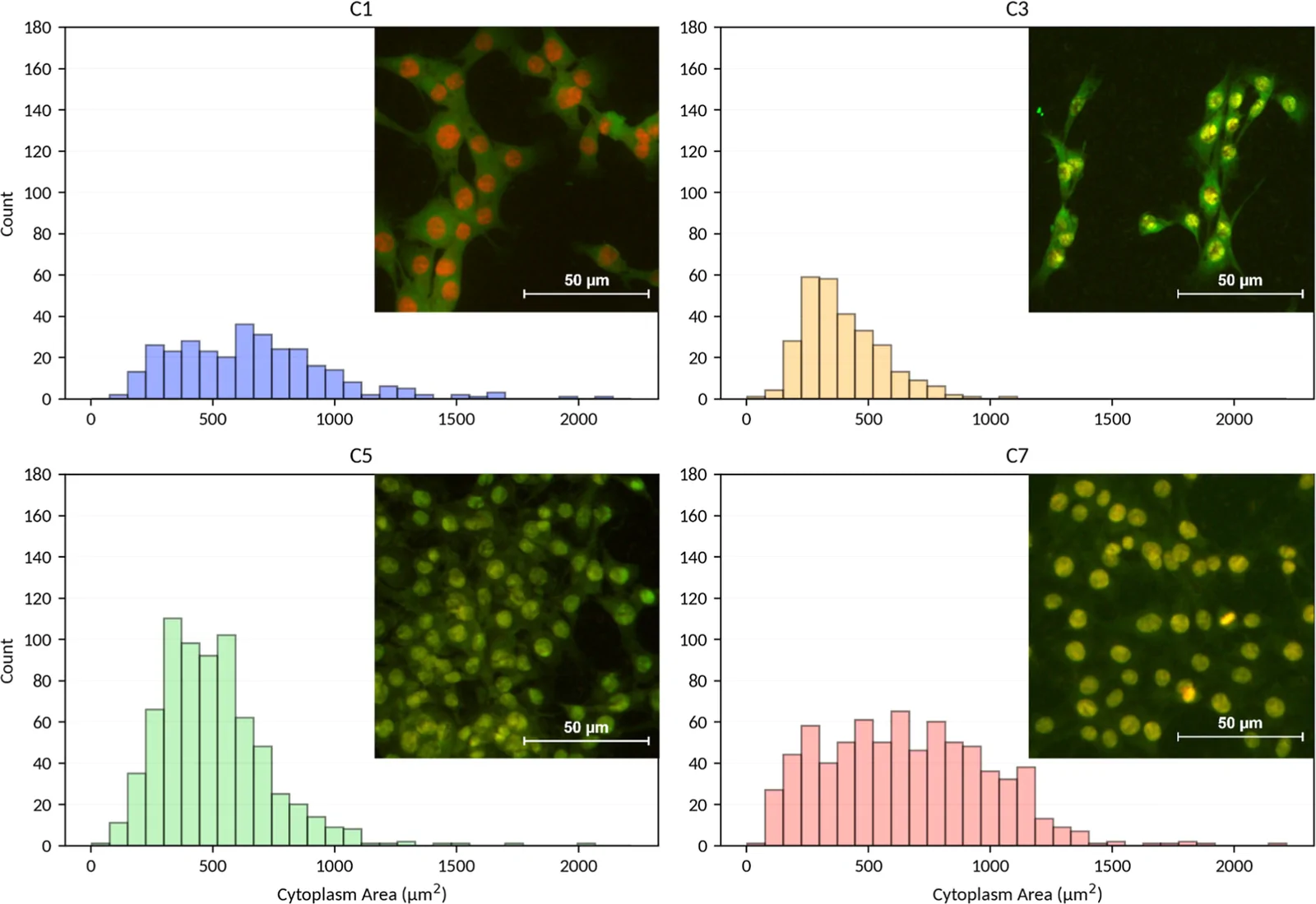
Cell (cytoplasm) area distribution across P3HBV films [C1, C3, C5, C7] for all samples. Histograms show the count of cells with the respective surface area for all regions, and the insets show representative images of the cells for every polymer sample.

Interestingly, improved roundness correlated with smoother surfaces characterized by fewer trenches and sharper features (low skewness and high kurtosis), presenting a contrast to the spreading behavior observed on rougher films. Table 1 provides an overview of AFM surface properties (roughness, kurtosis, and skewness) and cell roundness across P3HBV films C1–C7. The lower and upper confidence intervals for cell roundness (95 percentile) were observed to be C1: [0.57, 0.65], C3: [0.65, 0.72], C5: [0.65, 0.73], and C7: [0.70, 0.75]. On computing the Pearson correlation, we observe the cell roundness to have a strong negative correlation with skewness (*r* = −0.98, *p* = 0.02), indicating that films with lower skewness have higher roundness. Kurtosis has a strong positive correlation with cell roundness (*r* = 0.947, *p* = 0.05); therefore, a film with high kurtosis gives higher cell roundness. Voronoi entropy, 0 ≤ *S*_*v*_ ≤ 1.69, is a parameter that characterizes the orderliness of a set of points on a plane, with *S*_*v*_ = 0 for an ordered set and *S*_*v*_ = 1.690 ± 0.001 for a random set of points.^36,37^ It was used as the distribution metric of spatial organization Voronoi entropy remained largely consistent across all films, showing only faint correlations with thickness or surface features. We attribute this stability to the inherent biological variability of cell cultures and the sample size constraints of the study.

Next, we perform surface morphology analysis to rigorously quantify surface topological differences. We employed persistence homology, utilizing Wasserstein and Bottleneck distances to compare persistence diagrams derived from AFM data. These metrics provide a robust framework for a comparative analysis of surface features, where the 1-Wasserstein distance (shown in Figure 5B) explores that the intragroup comparisons revealed high topological consistency among films cast at the same concentration having similar thicknesses (e.g., low Wasserstein distances between replicates C1–S1, C1–S2, and C1–S3). However, as polymer concentration and film thickness increased, the intersample variability also rose; films with 7% concentration and maximum thickness (C7) exhibited significantly higher internal Wasserstein distances compared to the 1% (C1) group. This indicates that while higher concentrations produce rougher features and thicker films, they also introduce greater stochastic variability in surface heterogeneity. Although pore morphology may influence cell colonization, the correlation analysis indicates that pore descriptors alone do not fully account for the observed cellular behavior. Mean pore count showed a strong negative trend with cell density (*r* = – 0.946, *R* ^2^ = 0.895, *p* = 0.054), whereas mean pore area exhibited only a weak positive correlation (*r* = 0.302, *R* ^2^ = 0.091, *p* = 0.698). Because neither relationship reached statistical significance, these results suggest that pore characteristics should be considered together with other surface properties, such as roughness, topography, stiffness, and wettability, rather than as independent predictors of cell attachment.

**Figure 5:**
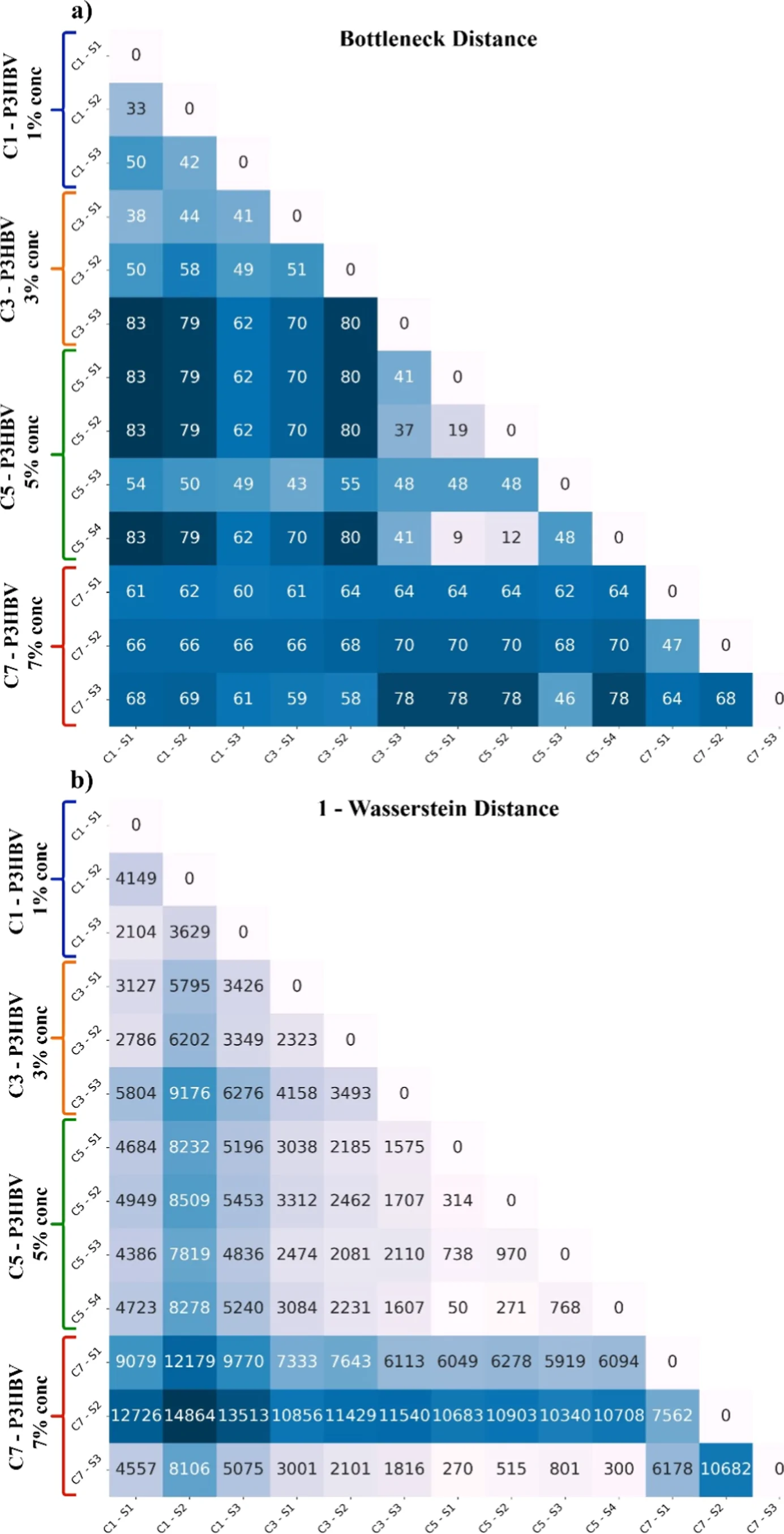
(A) Bottleneck distance and (B) 1-Wasserstein (earth mover’s) distance showing the topological similarity index between synthesized P3HBV films [C1, C3, C5, C7], where an increase in polymer concentrations is proportionally related to the increase in film thickness between generated surface features (i.e., film synthesized at C1 (1% concentration) is similar to that at C3 (3% concentration) but very far and distant from a film synthesized at C7 (7% concentration).

Bottleneck distance (shown in Figure 5A) analysis reinforced these findings by quantifying the maximum difference between the surface topological signatures.

Thin films from C1 with 1% concentration characterized by low roughness, low skewness, and low kurtosis (smooth surfaces with mild peaks) showed minimal topological deviation from one another; we observed topographical uniformity. In contrast, thick films from C7 with 7% concentration distinguished by high roughness and complex trench-ridge networks exhibited larger Bottleneck distances when compared to thin films. Consistent with theory, film pairs with similar morphologies yielded low Bottleneck distances, whereas comparisons between smooth (low concentration) and coarse (high concentration) films produced high distance values, validating the metric’s ability to distinguish varying degrees of surface complexity.

Next, data from height-threshold filtration lacunarity measures of 12 combinations of parametric setups were analyzed and studied relative to each film, and an overview of their surface spreading was observed. With the change in the number of threshold slices from 100, 500, to 1000, we noted an increase in fine detail that would otherwise have been missed in smaller threshold slices. The proportion of features being impacted was relatively the same across all films. Therefore, we decided to continue with results from 1000 threshold slices as the standard while comparing the results from 100 and 500 slices as well. With changes in box counting area (first and second order) and the surrounding neighborhood (4 and 8 neighbors), we observed that the behavior and features of the surfaces (i.e., holes, external lands, internal holes, and internal lands) remain the same, and the difference in these characteristics is only observed with an increase in the level of the threshold slices, i.e., with 1000 slices, the maximum level of detail within the surfaces is observed. Figure 6 provides an overview of all the features obtained from the surface after threshold analysis across 1000 slices with their mean and standard deviation.

**Figure 6:**
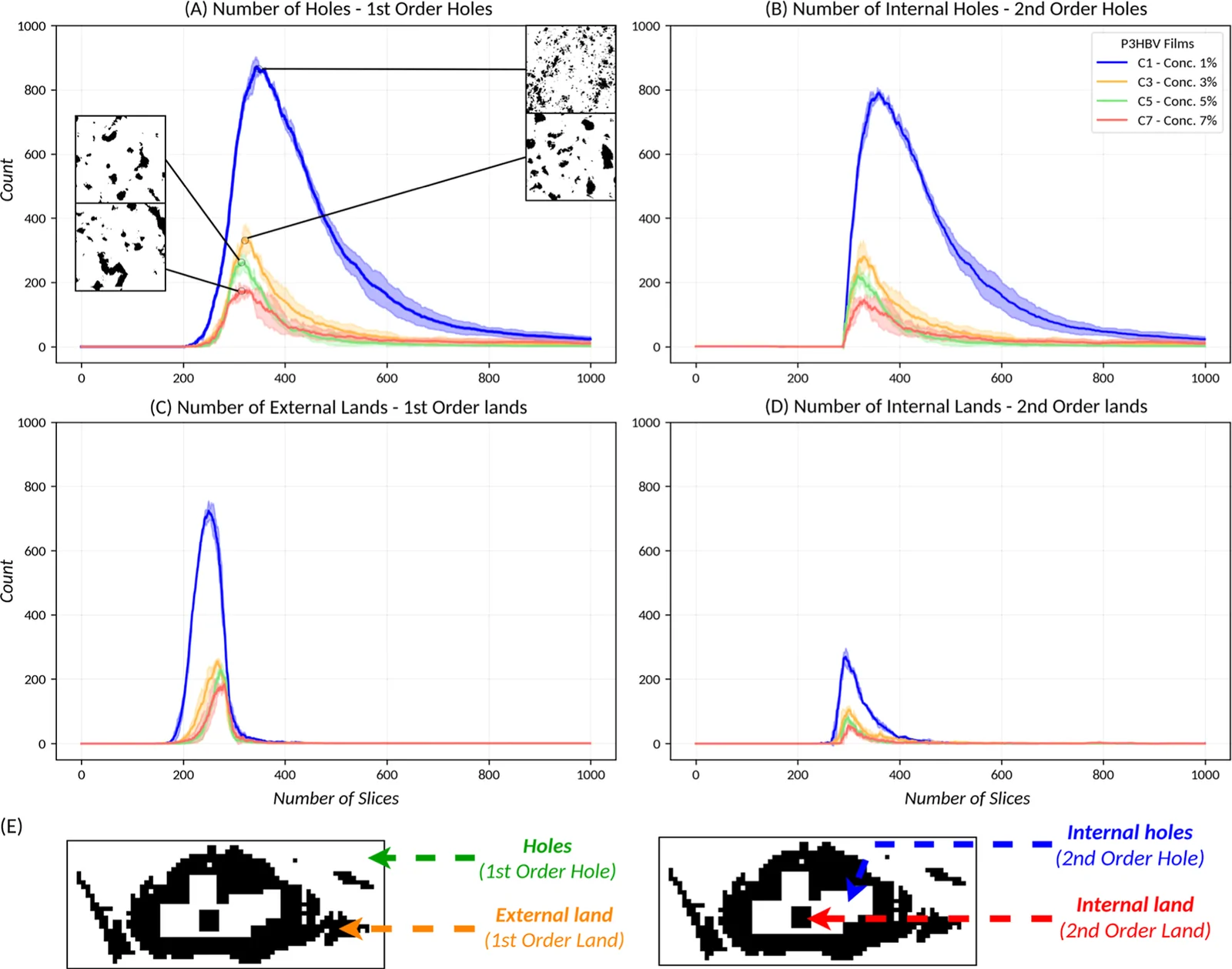
An overview of height-threshold filtration of P3HBV films [C1, C3, C5, C7] when analyzed with advanced relief analysis at 1000 slices and their average number of (A) holes, (B) internal holes, (C) external lands, and (D) internal lands with their standard deviation relative to the number of samples at each concentration. (E) A schematic overview of a selected region visualizing features of TRA, i.e., holes, internal holes, external lands, and internal lands.

Further, principal component analysis (PCA) was applied to the matrix of pairwise TDA-distance features to visualize dominant patterns of intersample variation in a reduced two-dimensional space. PC1 and PC2 therefore summarize the major variance structure of the distance-based representation rather than correspond directly to any single distance metric. Figure 7 shows the visualization of the dimensional space after PCA. We observe underlying similarity patterns as clusters of points, i.e., samples from (C1, C3) and (C5, C7) groups were packed mutually closer together, representing intragroup closeness, while intergroup distance represented between groups, reflecting surface difference between concentrations of the films.

**Figure 7:**
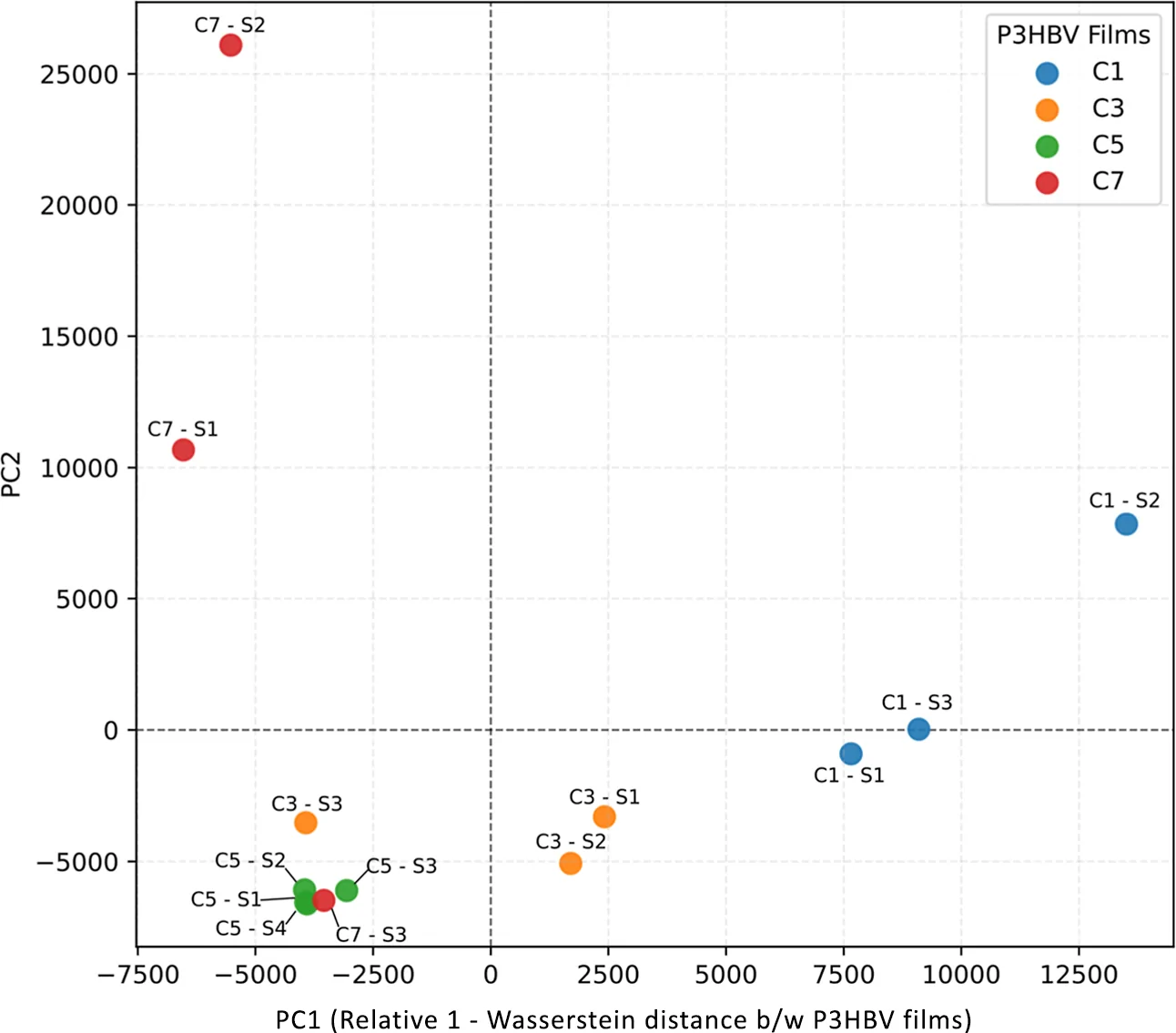
Visualized distribution of P3HBV C1, C3, C5, and C7 samples after PCA conversion representing the dissimilarity and mean variance between samples to achieve mutual surface features. PC1 represents the 1-Wasserstein distance between the P3HBV films, and PC2 represents the secondary distance of variance between them.

Using the SEM images of P3HBV, shown in Figure 2C, we segment and analyze the pore condition existing on the surface of these films. We use Cellpose Plus to perform this segmentation; however, due to the nature of pores being different from cells, we selected the area covered by pores and the count of pores as the descriptive metric of these images (Table S4 in Supporting Information shows extracted pore metrics for SEM images).

To enhance the interpretability of our results and comparability between films, we visualize a distribution of the pores on the surface relative to their frequencies of occurrence, as shown in Figure 8. At lower thickness, C1 and C3, there exists a higher number of small size pores (i.e., pores of small area), while at higher thickness, C5 and C7, we observe a gradual distribution with an increase in pore size across the latter end (i.e., pore size increases along with film thickness). Further, we explore the existence of correlation between cell growth manner and pore size, by comparing the pore state from SEM with the cell spreading and confluence from the fluorescence microscope and Cellpose Plus.

**Figure 8:**
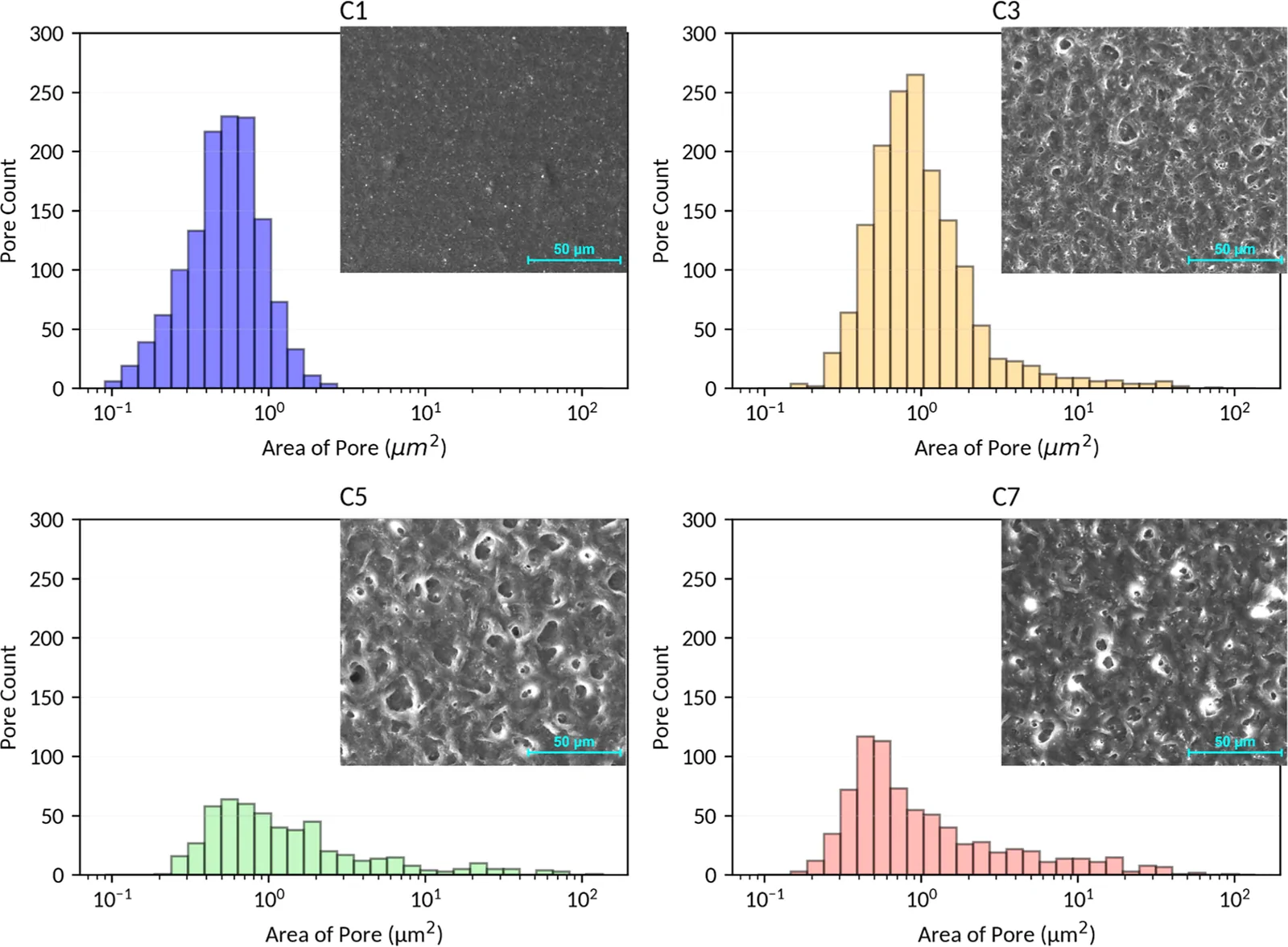
Log-scale distribution representing the average surface area of pores on the P3HBV films against the frequency of pore sizes for [C1, C3, C5, C7] films, respectively, as captured with SEM.

We combine the acquired results from AFM, SEM, fluorescence microscope, and their respective descriptive characteristics to investigate the collective relationship of cell growth and adhesion with film surface morphology. We also introduce lacunarity measures as a metric describing the layer-wise surface morphology as the integral of the number of components (holes or lands) over the number of thresholds computed as $A=\int_{L_{s}=0}^{n}Lds$ , where L is the surface features (holes, external lands, internal holes, and internal lands) integrated over the *L*_*S*_^th^ threshold level from 0 to *n* ^*th*^ threshold (corresponding to the maximum height), and ds is the step of threshold. Figure 9 presents a correlation matrix that combines derived metrics and characteristics to highlight correlations.

**Figure 9:**
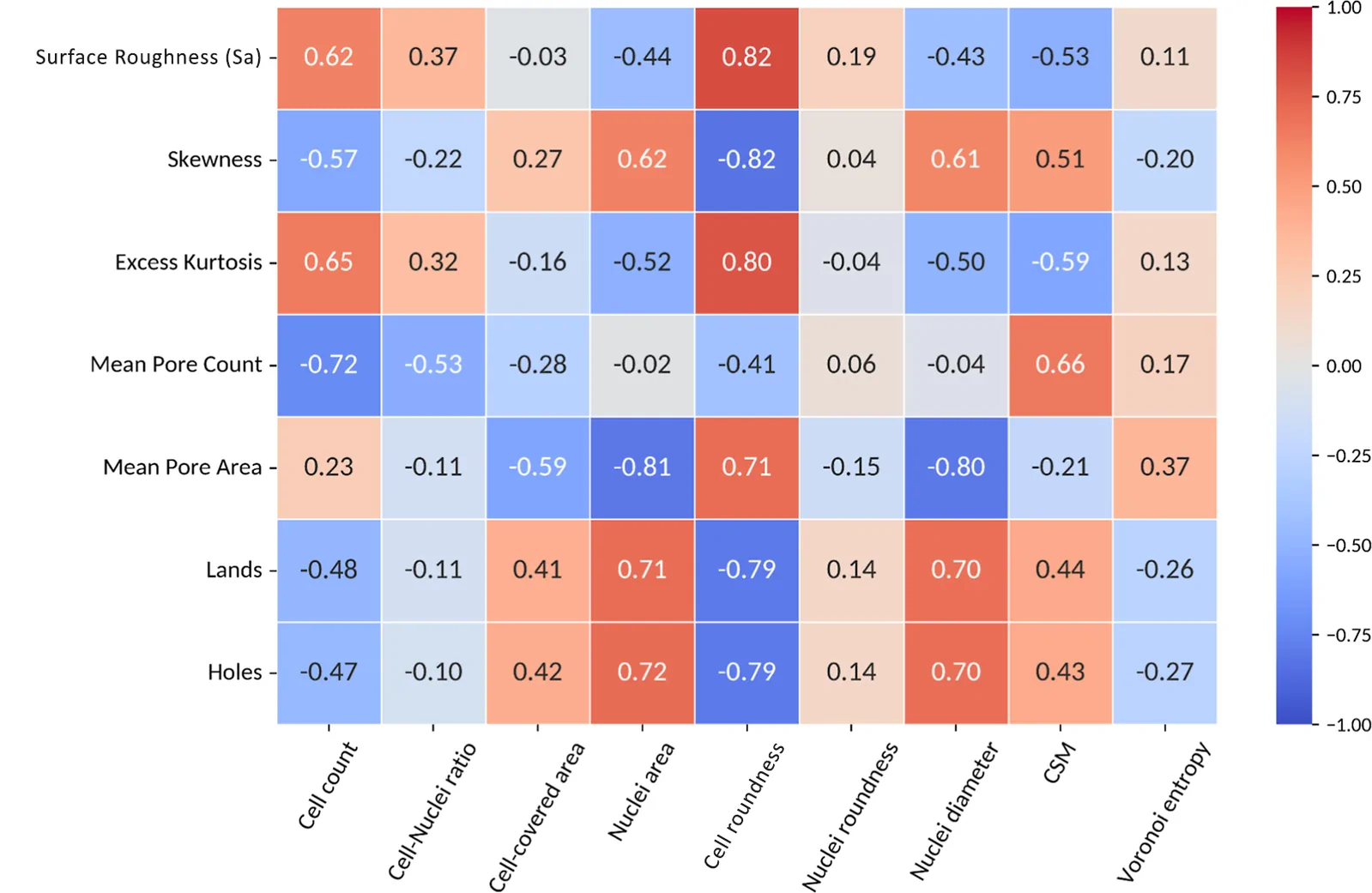
Correlation matrix depicting the inter-relation between measured surface morphology, pore characteristics, and thickness properties of P3HBV films when analyzed against the cell behavior metrics of C2C12 cells synthesized on corresponding P3HBV films relative to film thicknesses (C1, C3, C5, C7).

Relative to the cell growth for each film, we observe that with the increase of polymer concentration and film thickness, the surface morphology features tend to be more harsh (i.e., when observed via TDA analysis and AFM data, C1 P3HBV has low roughness and skewness, excess kurtosis, and a smooth surface with few trenches and ridges, compared to C7 P3HBV which has more coarse surface with higher roughness and skewness and excess kurtosis-ridges, trenches, and valleys). With an increase in surface roughness (S_a_), we observe a linear increase in cell count, with an increase in cell roundness at greater levels of surface roughness and a reduction in the area of nuclei, which means reduction of cell spreading and motility, while the skewness and excess kurtosis had inverse relations with the cell count and cell-covered area (i.e., with increased ridges, valleys, and troughs on the surface, the number of cells growing on the surface were observed to be higher on par with increasing thickness). This dependence is also observed within the lacunarity measure (height-threshold filtration) and integral component, where an increase in the number of holes reflecting surface features (e.g., peaks and troughs) negatively impacts the cell count in a given film (i.e., films with higher morphology components support greater cell growth). From SEM images, the number of pores on the film’s surface is considered as a feature that reflects parallel behavior with that of the lacunarity measure (i.e., an increase in pores reduces the number of cells grown on the film surface). The observed pore count in Table 1 and Figure 8 reflect the interconnection of material properties where films of lower thickness have a larger number of pores, and with the increase of thickness, fewer pores but with a larger area (greater in total volume) are formed. Figure S10 in Supporting Information shows a polar representation of the structure–property relationship between P3HBV solution concentrations and surface data from topological persistence barcodes (Wasserstein distance) against cell count, describing a metric-point state space.

We observe a concentration-driven transition in surface height distribution, where thin films (C1 and C3 of thicknesses 13–30 μm) are relatively smooth (with low S_a_) and have peak-dominated surfaces (i.e., S_ku_: ∼ 0), whereas thicker films (C5–C7 of thickness 45–76 μm) progressively shift toward valley–trench-enriched surfaces (i.e., S_sk_ < 0), reflecting sharp-edged morphology (i.e., higher kurtosis). The transition from positive to negative skewness occurred monotonically with increasing polymer concentration, and no nonmonotonic behavior was observed for the intermediate concentrations. Although solvent casting does not allow complete control over solvent evaporation dynamics and the exact formation of local surface features, resulting in some variability between independently prepared samples, the overall concentration-dependent trend in surface morphology remained consistent at the group level. This transitional feature is characteristic for P3HBV casted films, where increasing the solution concentration can amplify mesoscale instabilities during solvent evaporation (e.g., local flow, shrinkage, and phase-separation-like patterning), producing deeper depressions and more pronounced ridge–trench networks on the air-exposed side used for cell culture. With TDA metrics, we acquire independent morphology statistics that reflect group-wise and sample-wise multiscale topology, i.e., films produced at the same concentrations exhibit high intragroup similarity (with low distances among replicates) and greater intergroup distances with an increases in P3HBV concentration, therefore indicating a systematic morphological “drift” rather than random roughening.

With Cellpose Plus, we observe surface morphology changes that influence biological development through quantifiable metrics such as cell count and cell density that strongly increase from [C1, C3] to [C5, C7], reflecting improved early attachment on thicker and rougher substrates. For cells with an average diameter of about 25–30 μm, the thickness of such film was about 45–76 μm. Such enhancement is broadly aligned with established biomaterial principles that cell adhesion, proliferation, and migration are sensitive to micro- and nanoscale topography through changes in protein adsorption, focal-adhesion maturation, and cytoskeletal organization, where moderately complex surfaces can provide more anchoring opportunities and reduce effective cell detachment under handling. We observe the coupled effect of negative skewness (S_sk_) (trenches/valleys) and feature width (S_ku_—kurtosis) that describes surface geometry associated with an increase in attachment and proliferation. Therefore, confirming concave features that promote mechanical interlocking and stable nascent adhesion during the early cell-surface contact interactions phases. The cell spreading (calculated via the roundness metric) and Voronoi entropy were relatively constant through an increase in surface thickness and support the prospect of early response that is dominated by adhesion/spreading heterogeneity at the single-cell scale rather than by large changes in spatial packing over the analyzed field of view.

SEM-based pore metrics allow us to quantify larger regions where proportional increases in pore size and film thickness were observed. Although pores in the film surface structure increase its total surface area and contribute to increased cell colonization density per film unit area and its volume, our results indicate that the pore descriptors used in this study alone may not be sufficient to fully explain cellular trends; therefore, combining SEM and AFM metrics within a hierarchical topography framework can integrate cellular behavior across multiple scales. This is consistent with the broader idea that cells respond to a spectrum of features—from nanometer-scale roughness, which influences protein layer organization, to micrometer-scale invaginations, impacting cell body placement and processes elongation.

We demonstrate that TDA-based persistent distances of barcodes (I-Wasserstein and bottleneck) and TRA descriptors (holes, islands, etc.) can serve as morphological coordinates that reveal geometric differences in the relative state space (shown in Figure 7 with PCA) when conventional parameters (Sa, Ssk, and Sku) are insufficient or ambiguous. For example, a higher P3HBV (C7) concentration and thickness increase roughness as well as intragroup heterogeneity (i.e., larger intragroup Wasserstein distances). Therefore, the synthesis of thicker and rougher films can introduce stochasticity that must be considered in cases where film reproducibility is a crucial factor. TDA provides a structured comparison of whole surface characteristics and offers a quantitative bridge between AFM images and statistical measurement results.

Overall, we observe that thicker P3HBV films produced from higher casting concentrations generate a hierarchical, trench-enriched surface with coarse morphology that enhances early potent growth of C2C12 attachment and proliferation. The cell growth was observed to be uniformly destributed, with clusters (C1, C3) and (C5, C7) showing high intracluster similarity and a major intercluster difference, as reflected by surface characteristics. Specifically, the confluence was 14 ± 6% for C1, 8 ± 3% for C3, 24 ± 9% for C5, and 34 ± 19% for C7. Our results provide a data-driven pipeline to combine and quantify data from AFM, SEM, and fluorescence microscopy for statistical analysis and morphology-based PHA group TDA approaches. Further, the results support a rational pathway for designing PHA-based biomaterial interfaces: selecting processing conditions that yield not only higher roughness but also an appropriate height distribution (negative skewness with suitably broad/connected features) while monitoring topological similarity/variability to ensure reproducibility across batches.

## 4 Conclusions

This work presents results of a bulk–surface–biointerface relationship experiment for casted P3HBV films of increasing polymer concentrations (1%, 3%, 5%, and 7%), yielding progressively thicker substrates with systematically evolving surface morphology across nano- to microscales and adhesive cell growth and proliferation features on respective surfaces.

AFM-based height-roughness statistics—surface roughness, skewness, and kurtosis, analyzed with TRA (lacunarity measure)—demonstrates a concentration-driven transition from smooth to coarse relief regime characterized by high roughness and a shift from peak-dominated to trench/valley-enriched height distributions. The persistence barcodes derived from TDA and distances (Wasserstein and Bottleneck) corroborate acquired results and explainability of intragroup and intergroup morphological consistency versus film thickness increases. C2C12 myoblasts as a model adhesive culture cultured on the respective surfaces exhibit enhanced early colonization on thicker and coarser films, with cell count, density, roundness, cell/nucleus size ratio, etc., as cell morphology metrics, derived from Cellpose Plus reflecting independent cell grow on film surfaces. Scanning electron microscopy-based pore analysis further contextualizes the interface by revealing concentration-dependent pore-size distributions and confirms mesoscale porosity contribution to the observed cell response within this hierarchical topography framework. With a comprehensive statistical analysis with AFM metrics, topology, and morphology aware surface distances (TRA and TDA), SEM descriptors, and cell behavior metrics, we present structure–property relational visualizations to map how naturally engineered P3HBV surface morphology regulates myoblast adhesion. Our results identify the coarse, porous, and trench-enriched multiscale topology of the films produced at higher polymer concentrations as a crucial factor for improving early cell attachment on P3HBV biointerfaces and provide transferable analytics for optimization of PHA-based surfaces in cell-interactive biomedical coatings and tissue-engineering platforms. Potentially, we may apply this tool to every human body cell type in frames of new polymeric implant development.

## Supplementary Material



## Data Availability

The data used in this experiment consisting of atomic force microscope (AFM), scanning electron microscope (SEM), fluorescence microscope, results of topological data analysis (TDA), and threshold relief analysis (TRA) is available for open access on Zenodo (doi.org/10.5281/zenodo.19043048)

## Footnotes

1 Gwyddion toolbox (gwyddion.net) last accessed 19–03–2026.

2
https://github.com/ITMO-MMRM-lab/Cellpose_plus.

## Notes

Atomic force microscope (AFM) scans of 1, 3, 5, and 7% polymer concentration thicknesses; AFM-derived morphological metrics; TDA-derived persistence barcodes and entropy metrics; TRA results of thresholding over box counting, thresholds, and neighborhoods; SEM images of all scanned areas for pore-size analysis and pore distribution metrics; fluorescence microscope images of C2C12 myoblast cells and cell metrics derived from the Cellpose Plus toolbox; and polar representation of structure–property relationship (PDF)
