# Supplementary material for: Integrative Infochemistry of Polyhydroxyalkanoate Films: Multimodal Data, Topological Tuning, and Cell–Surface Interfaces
Source: Langmuir. 2026 Aug 22;42(35):25517–31. doi: 10.1021/acs.langmuir.6c02572 (PMC13564920; doi:10.1021/acs.langmuir.6c02572)
Supplement: Supplementary file 1 [file la6c02572_si_001.pdf]

Supporting Information

Integrative Infochemistry of Polyhydroxyalkanoate Films: Multimodal Data, Topological Tuning, and Cell-Surface Interfaces

Fares D. E Ghorabe[1], Ashish T.S. Ireddy[1], Pavel S. Zun[1], Israel A. Huaman[1], Sviatlana A. Ulasevich[1], Aleksandr S. Aglikov[1], Dmitry A. Kozodaev[2], Tatiana G. Volova[3,4], Ekaterina I. Kessler Shishatskaya[3], Michael Nosonovsky[1,5], Ekaterina V. Skorb[1,]*

[1]Infochemistry Scientific Centre, ITMO University, 9 Lomonosova St. 191002, St. Petersburg, Russia

[2]NT-MDT BV, 7335 Apeldoorn, The Netherlands

[3]Siberian Federal University, 79 Svobodnyi Av., Krasnoyarsk - 660041, Russia

[4]Institute of Biophysics SB RAS, Federal Research Center "Krasnoyarsk Science Center SB RAS", Akademgorodok, 50/50, 660036 Krasnoyarsk, Russia

[5]University of Wisconsin-Milwaukee, Milwaukee, WI 53217 USA

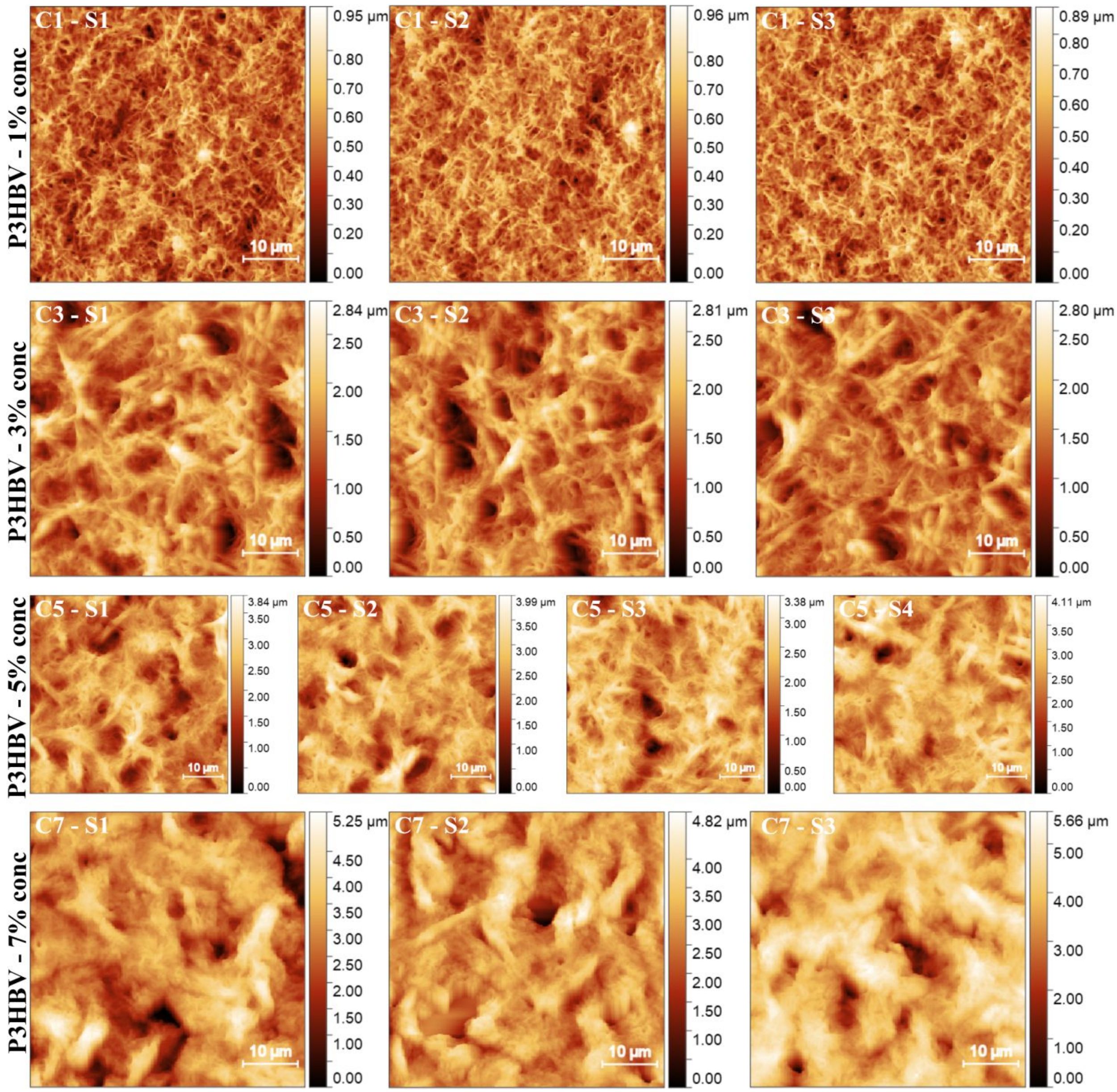

Figure S1. P3HBV films of C1, C3, C5, C7 thicknesses scanned and visualized using atomic force microscopy.

Table S1: Atomic force microscopy morphology metrics of individual P3HBV of films C1, C3, C5, C7 thicknesses

#	*Name*	**P3HBV *Conc***	S_a	S_q	S_{sk}	S_{ku}
1.	C1 - S1	1%	89.96	113.41	0.12	0.13
2..	C1 - S2		88.71	111.79	0.02	0.13
3.	C1 - S3		86.69	108.37	0.03	-0.05
4.	C3 - S1	3%	312.42	401.55	-0.38	0.46
5.	C3 - S2		312.98	397.29	-0.37	0.25
6.	C3 - S3		270.98	348.62	-0.16	0.51
7.	C5 - S1	5%	390.94	496.74	-0.14	0.27
8.	C5 - S2		393.08	504.78	-0.41	0.53
9.	C5 - S3		343.07	441.43	-0.64	1
10.	C5 - S4		389.69	493.3	-0.42	0.84
11.	C7 - S1	7%	609.41	793.49	-0.45	0.72
12.	C7 - S2		459.74	587.01	-0.21	0.5
13.	C7 - S3		610.05	787.44	-0.72	0.87

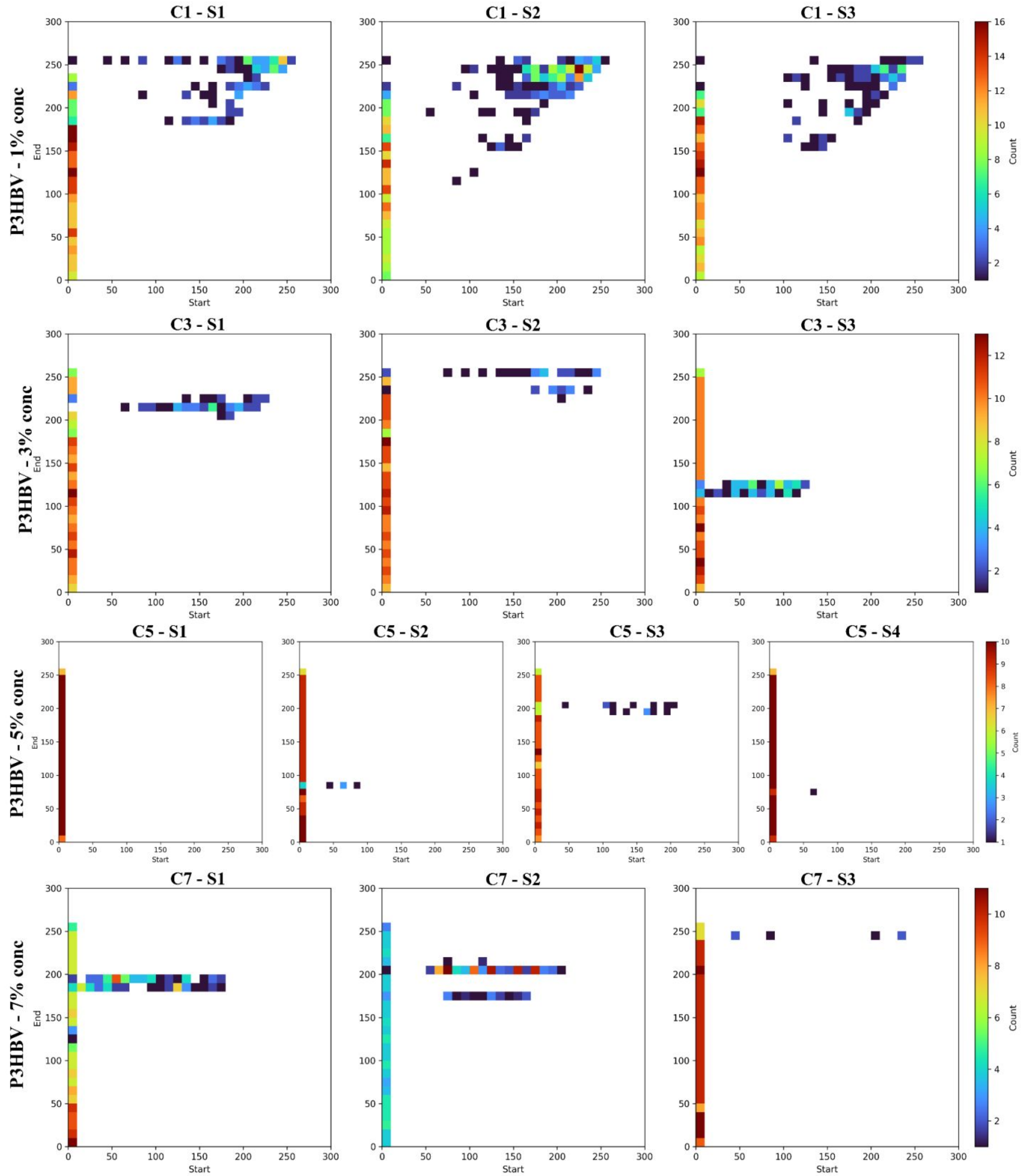

Figure S2. Persistence barcodes of P3HBV films computed from Topological Data Analysis approach (TDA)

Table S2. TDA **H0** entropy metrics for P3BHV [C1, C3, C5, C7] films

#	P3HBV Films	$H0_n$	$H0_L$	$H0_{Lavg}$	$H0_{Entropy}$	$H0_{MaxL}$	$H0_{MinL}$	$H0_{VarL}$
1	C1-S1	256	29607.23	115.65	5.37	255	1	4106.19
2	C1-S2	256	28241.55	110.32	5.38	254	1	3526.33
3	C1-S3	256	28675.85	112.02	5.38	255	1	3652.43
4	C3-S1	255	30156.97	118.26	5.34	255	1	5004.06
5	C3-S2	255	31034.49	121.7	5.35	255	1	4870.77
6	C3-S3	255	31928.24	125.21	5.33	255	1	5791.03
7	C5-S1	255	32891	128.98	5.35	255	1	5420.65
8	C5-S2	255	32501.31	127.46	5.34	255	1	5664.62
9	C5-S3	255	32135.58	126.02	5.34	255	1	5467.89
10	C5-S4	255	32816.88	128.69	5.35	255	1	5471.68
11	C7-S1	255	28898.24	113.33	5.27	255	0.22	6261.91
12	C7-S2	255	31018.62	121.64	5.33	255	1	5488.15
13	C7-S3	255	32362.65	126.91	5.35	255	1	5418.55

Table S3. TDA **H1** entropy metrics for P3BHV [C1, C3, C5, C7] films

#	**P3HBV Films**	$\mathbf{H1_n}$	$\mathbf{H1_L}$	$\mathbf{H1_{Lavg}}$	$\mathbf{H1_{Entropy}}$	$\mathbf{H1_{MaxL}}$	$\mathbf{H1_{MinL}}$	$\mathbf{H1_{VarL}}$
1	C1-S1	124	4812.53	38.81	4.38	202.48	0.14	1612.06
2	C1-S2	243	9218.41	37.94	5.17	158.64	0.18	1003.06
3	C1-S3	94	3271.86	34.81	4.18	123.16	0.62	948.56
4	C3-S1	50	2754.58	55.09	3.67	144.9	2.94	1387.8
5	C3-S2	35	2004.99	57.29	3.29	175.33	1.4	1781.8
6	C3-S3	64	2781.25	43.46	3.96	100	0.79	697.73
7	C5-S1	0	0	0	0	0	0	0
8	C5-S2	5	114.02	22.8	1.5	37.41	6.41	97.07
9	C5-S3	15	795.16	53.01	2.36	154.4	0.21	1789.45
10	C5-S4	1	17.32	17.32	0	17.32	17.32	0
11	C7-S1	123	13844.55	112.56	4.71	181.26	10.55	2142.01
12	C7-S2	257	20959.49	81.55	5.4	158.56	1.42	1781.89
13	C7-S3	6	593.04	98.84	1.33	196	1.16	7253.67

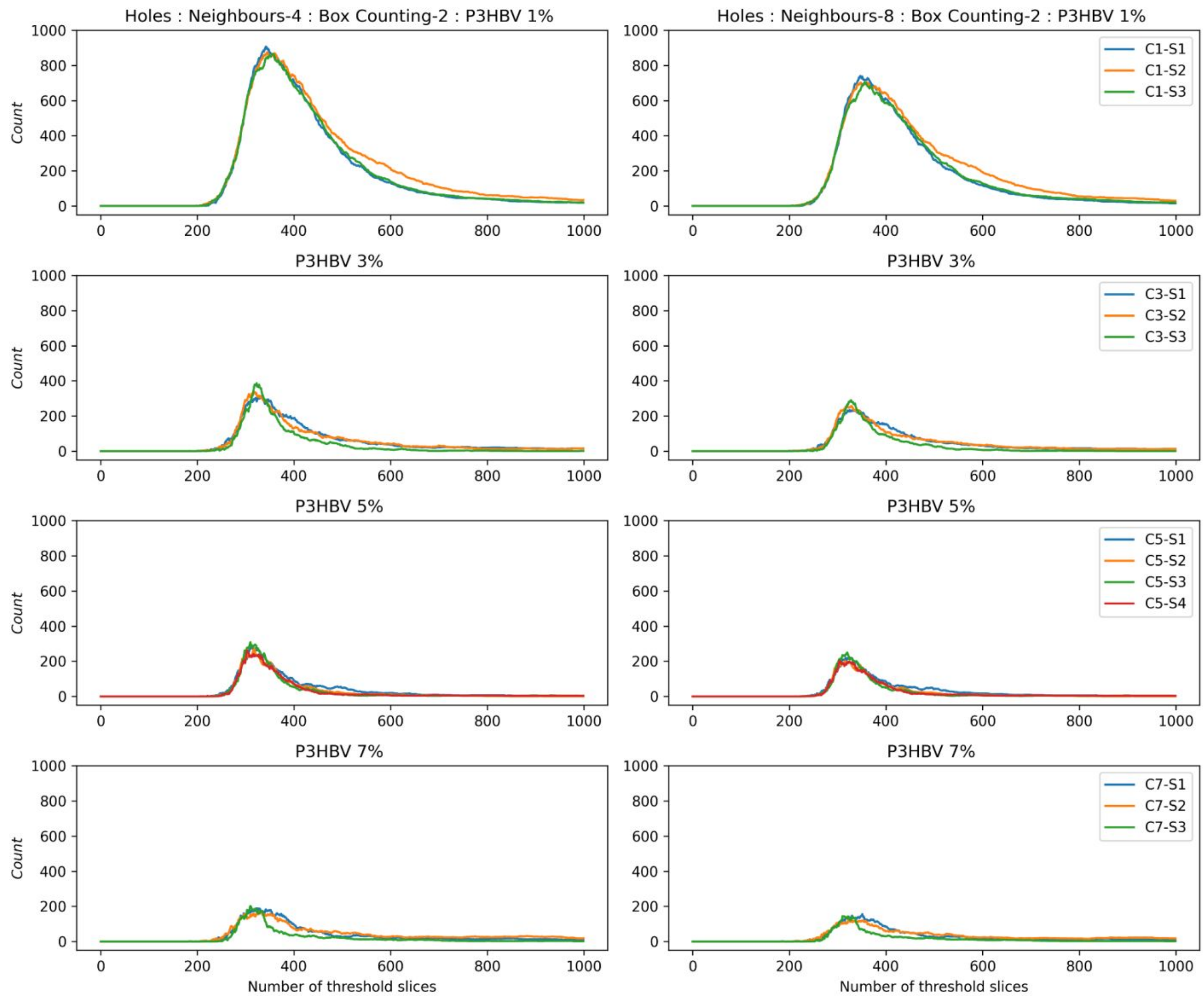

Figure S3. The number of holes observed by TRA over 1000 threshold slices with 2nd order box counting approach for C1, C3, C5, C7 P3HBV films in (Left column) 4-neighbourhood and (Right column) 8-neighbourhood areas

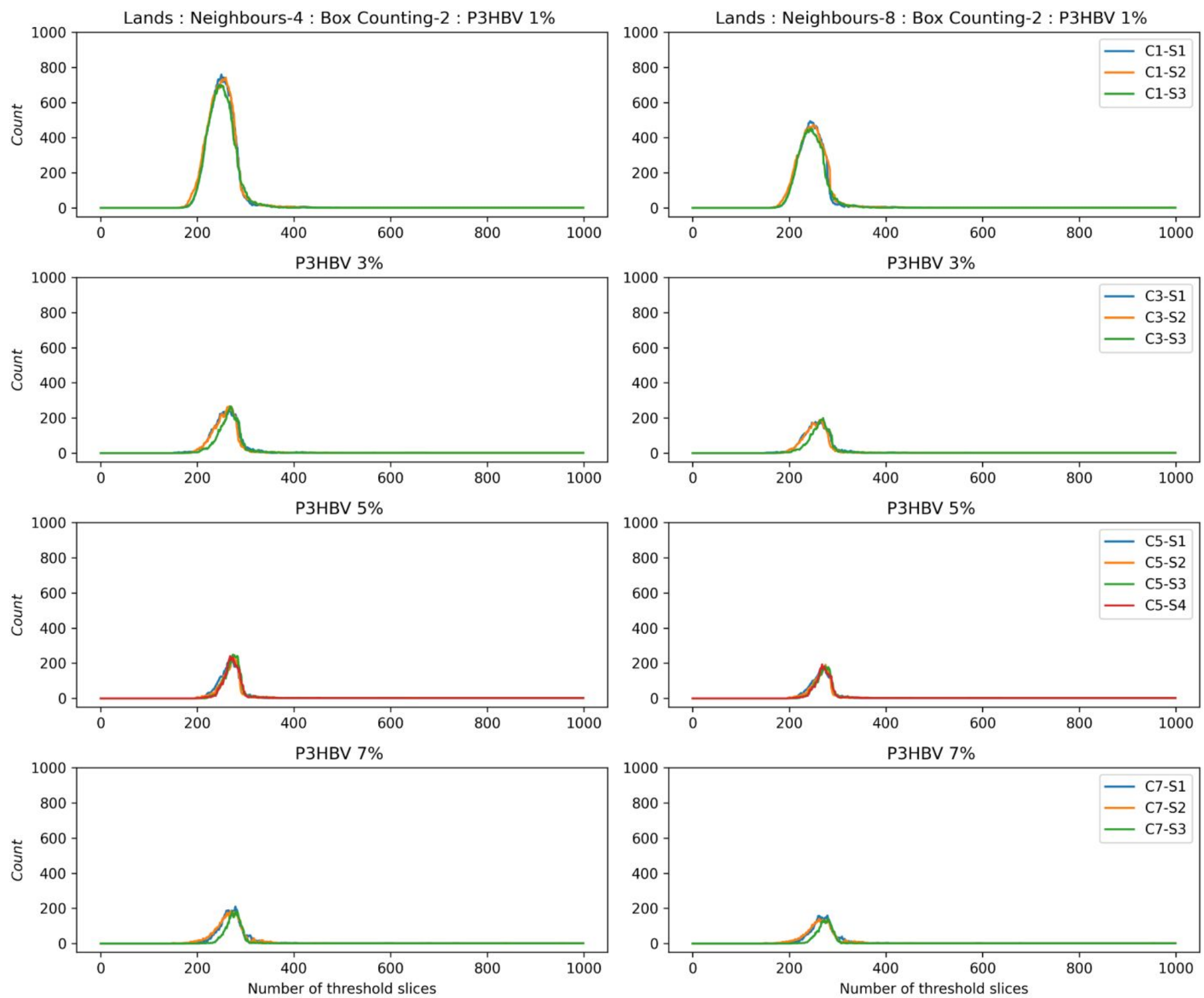

Figure S4. The number of **lands** observed by TRA over 1000 threshold slices with 2nd order box counting approach for C1, C3, C5, C7 P3HBV films in (Left column) 4-neighbourhood and (Right column) 8-neighbourhood areas.

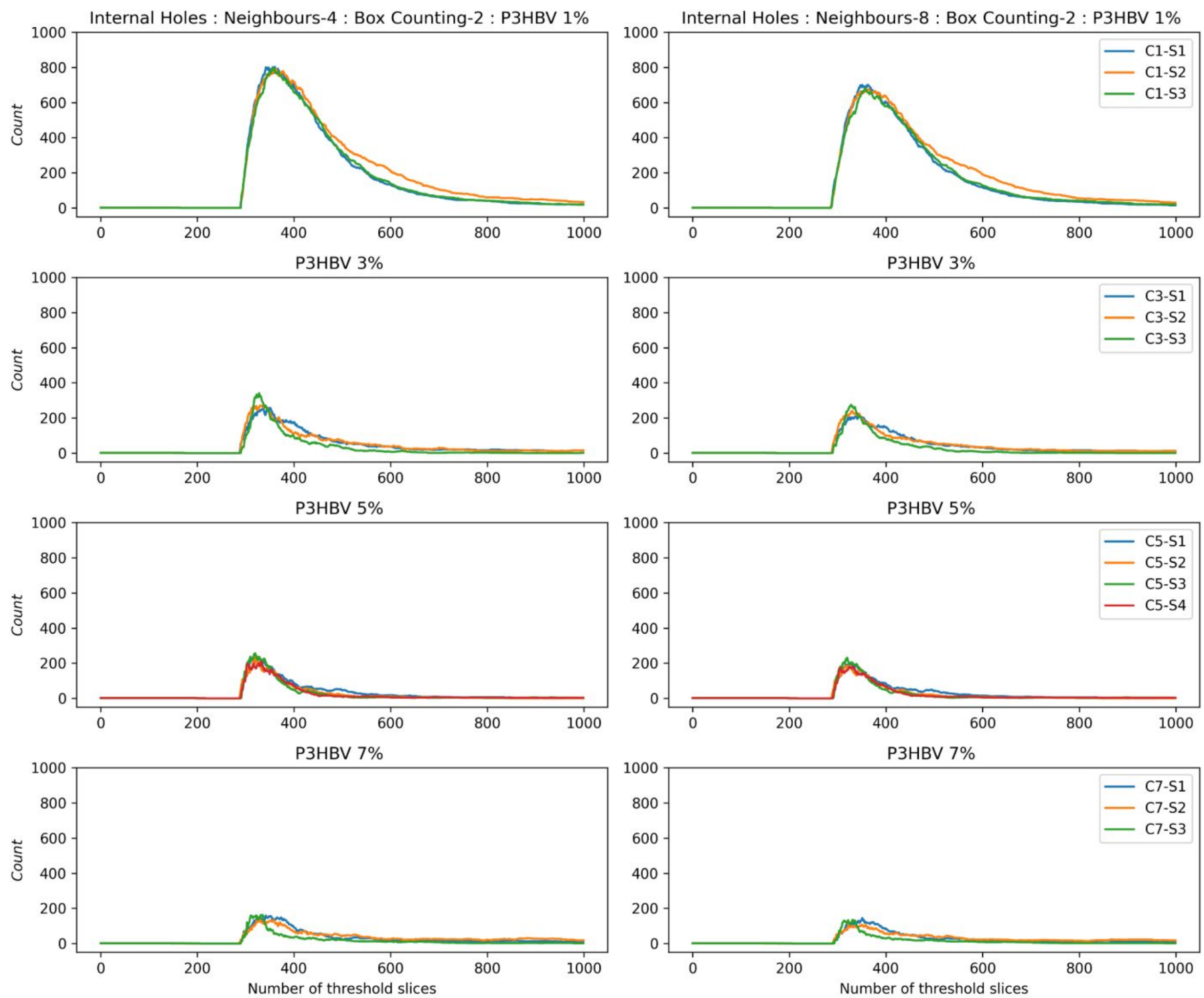

Figure S5. The number of **internal holes** observed by TRA over 1000 threshold slices with 2nd order box counting approach for C1, C3, C5, C7 P3HBV films in (Left column) 4-neighbourhood and (Right column) 8-neighbourhood areas

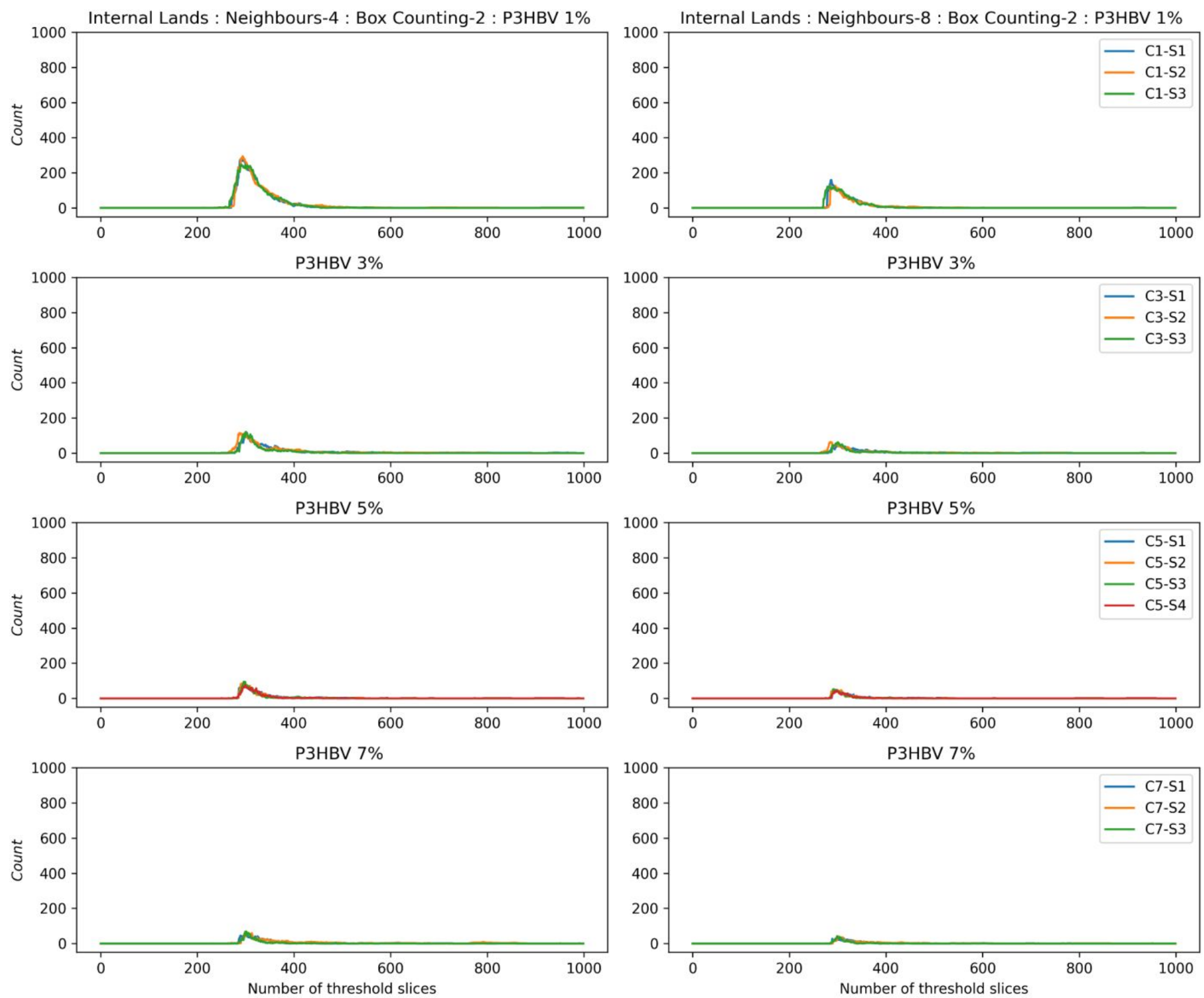

Figure S6. The number of **internal lands** observed by TRA over 1000 threshold slices with 2nd order box counting approach for C1, C3, C5, C7 P3HBV films in (Left column) 4-neighbourhood and (Right column) 8-neighbourhood areas.

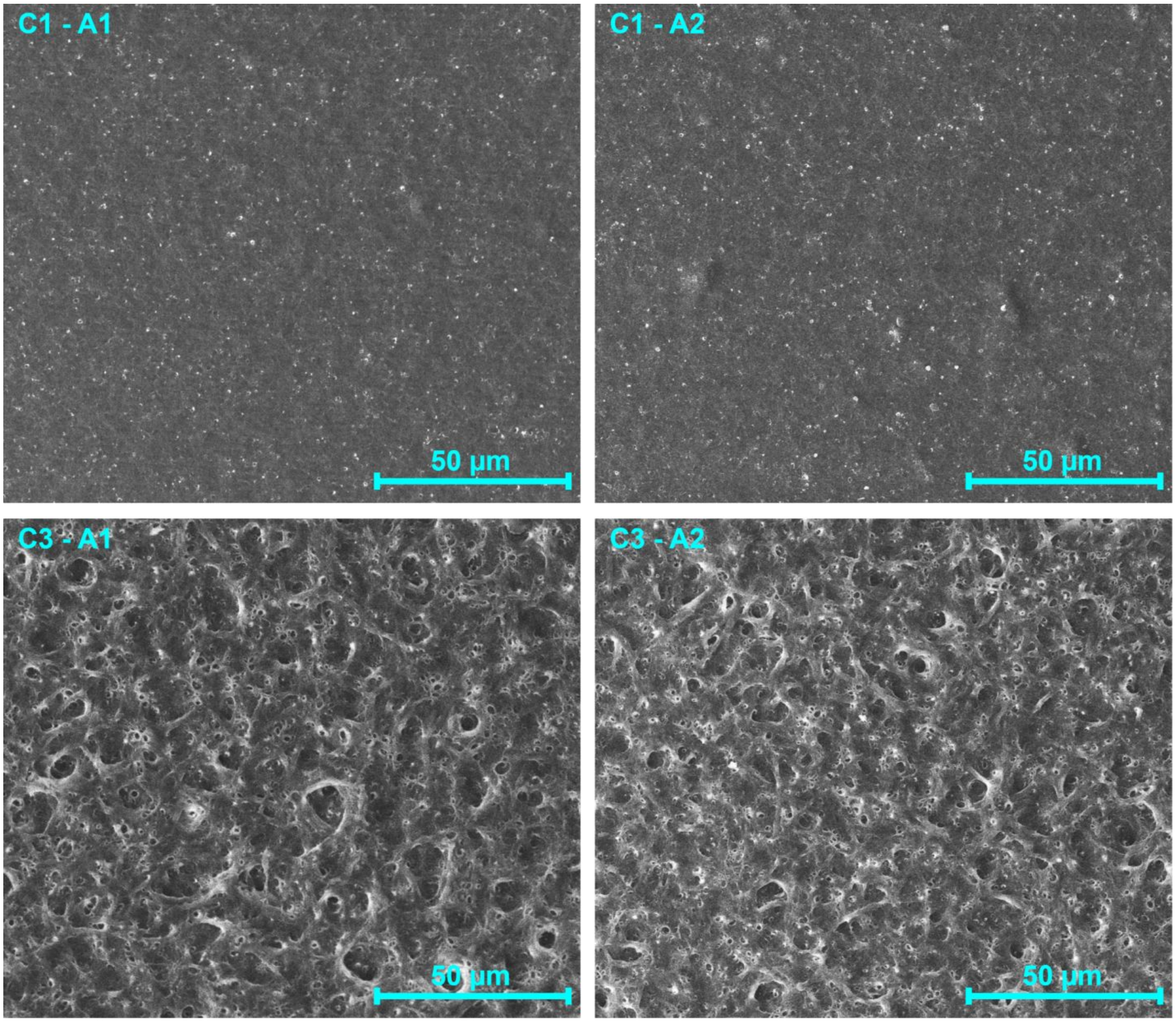

Figure S7. Scanning electron microscope (SEM) images of P3HBV films **C1** and **C3** over two unique areas

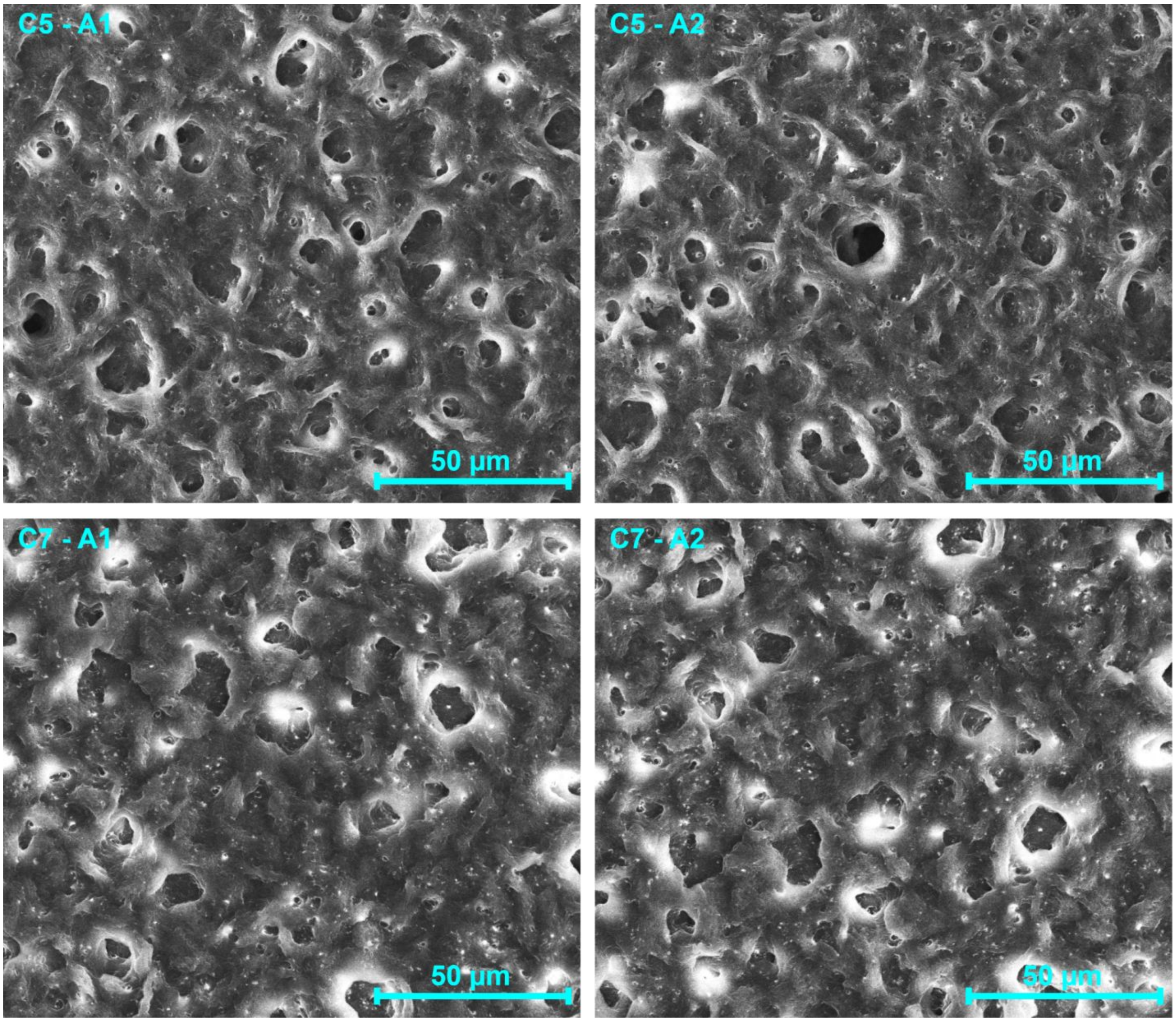

Figure S8. Scanning electron microscope (SEM) images of P3HBV films **C5** and **C7** over two unique areas

Table S4. Pore distribution metrics computed on the SEM images of P3HBV films C1, C3, C5, C7 over two areas (A1, A2) for images of scale 30µm x 30µm area.

P3HBV Film	***Image Area***	***Number of Pores***	***Total Pore area (µm²)***	***Pore Area per µm²***	***Pore density in scan area***
C1	A1	642	374.98	0.046	0.584
	A2	657	407	0.050	0.619
C3	A1	895	1359.78	0.166	1.519
	A2	673	1288.63	0.157	1.915
C5	A1	252	1281.62	0.157	5.086
	A2	278	859.11	0.105	3.090
C7	A1	342	1194.63	0.146	3.493
	A2	433	1063.2	0.130	2.455

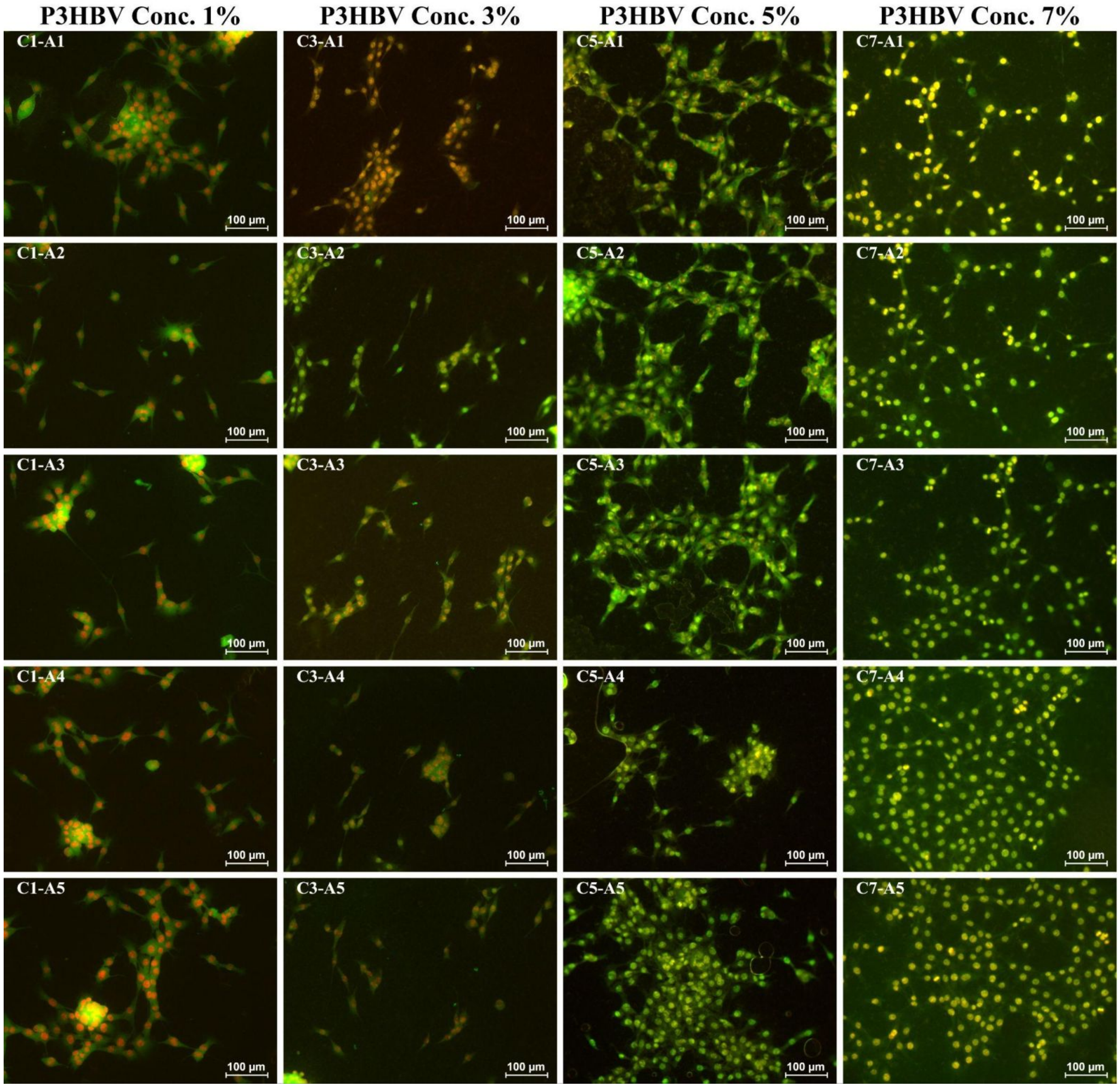

Figure S9. Fluorescence microscopy scan images of C2C12 Myoblast cells synthesized on P3HBV films of concentration C1, C3, C5, C7

Table S5: Myoblast C2C12 cell metrics extracted from fluorescence microscope images via Cellpose plus toolbox.

P3HBV Conc	Sample Area	Cell Count	Cell Cytoplasm ratio	Average Cytoplasm Area	Average Nuclei Area	Cytoplasm roundness	Nuclei roundness	Nuclei Diameter	CSM	Voronoi Entropy
C1	1	87	4.56	695.02	156.19	0.6	0.96	7.05	0.15	1.49
	2	35	3.48	611.21	171.29	0.58	0.93	7.38	0.15	1.47
	3	44	2.86	530.08	179.33	0.66	0.93	7.56	0.21	1.68
	4	59	3.59	676.97	187.89	0.58	0.93	7.73	0.17	1.5
	5	86	3.99	693.1	174.4	0.63	0.94	7.45	0.17	1.54
C3	1	82	3.2	388.03	124.27	0.7	0.94	6.29	0.14	1.61
	2	73	2.96	410.21	141.25	0.66	0.92	6.71	0.18	1.58
	3	50	3.44	418.27	129.32	0.66	0.91	6.42	0.15	1.66
	4	42	3.17	344.76	116.55	0.72	0.94	6.09	0.2	1.73
	5	35	3.09	395.03	129.76	0.66	0.94	6.43	0.18	1.58
C5	1	145	3.93	526.97	147.19	0.67	0.91	6.84	0.14	1.5

	2	160	4.04	518.12	143.26	0.68	0.92	6.75	0.15	1.56
	3	142	3.67	522.28	151.91	0.67	0.92	6.95	0.13	1.68
	4	56	4.49	496.61	119.13	0.67	0.93	6.16	0.14	1.61
	5	206	3.18	452.64	149.08	0.75	0.93	6.89	0.13	1.5
C7	1	104	3.66	497.25	136.01	0.69	0.95	6.58	0.16	1.62
	2	106	3.98	549.34	136.59	0.72	0.95	6.59	0.13	1.56
	3	121	3.93	532.74	141.05	0.75	0.94	6.7	0.15	1.67
	4	216	4.6	764.2	163.81	0.73	0.95	7.22	0.14	1.55
	5	197	4.81	773.27	162	0.72	0.95	7.18	0.12	1.44

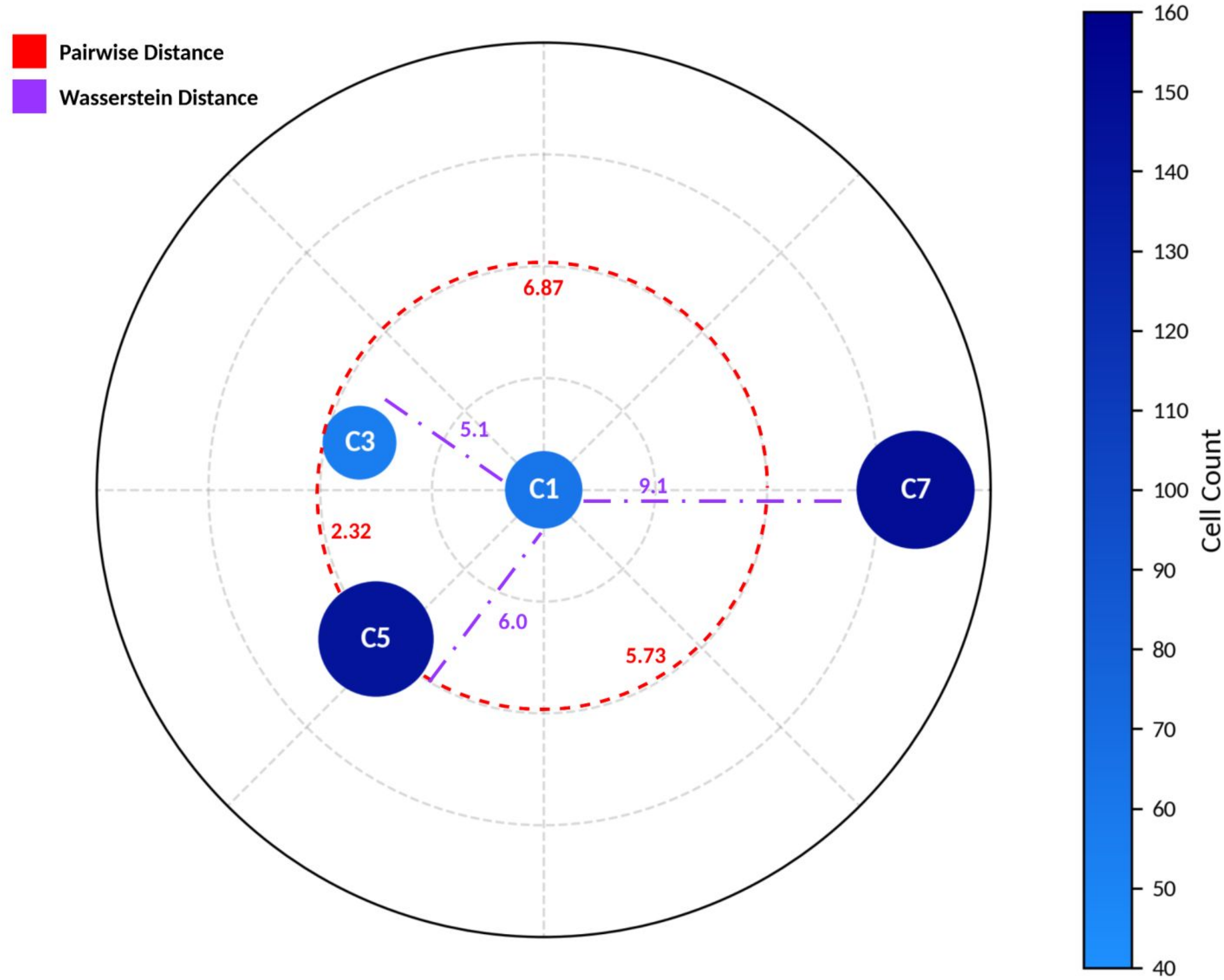

Figure S10: A polar representation of the structure-property relationship between topological data surface data from persistence barcodes (Wasserstein distance) against cell count where P3HBV C1 is considered the baseline and compared against C3, C5, C7 samples; The distance to each point represents the Wasserstein distance from C1; The angle represents the pair-wise intra distance between C3-C5, C5-C7. C7-C3; The size of each point represents the number of cells grown on each film of C1, C3, C5, C7 thicknesses.

The data used in this experiment consisting of atomic force microscope (AFM), Scanning electron microscope (SEM), fluorescence microscope, results of topological data analysis (TDA) and threshold relief analysis (TRA) is available for open access at doi.org/10.5281/zenodo.19043047
